# Cladribine treatment improves cortical network functionality in a mouse model of autoimmune encephalomyelitis

**DOI:** 10.1186/s12974-022-02588-7

**Published:** 2022-11-08

**Authors:** Christina B. Schroeter, Leoni Rolfes, K. S. Sophie Gothan, Joel Gruchot, Alexander M. Herrmann, Stefanie Bock, Luca Fazio, Antonia Henes, Venu Narayanan, Steffen Pfeuffer, Christopher Nelke, Saskia Räuber, Niklas Huntemann, Eduardo Duarte-Silva, Vera Dobelmann, Petra Hundehege, Heinz Wiendl, Katharina Raba, Patrick Küry, David Kremer, Tobias Ruck, Thomas Müntefering, Thomas Budde, Manuela Cerina, Sven G. Meuth

**Affiliations:** 1 Department of Neurology, Medical Faculty, Heinrich-Heine-University Düsseldorf, Moorenstraße 5, 40225 Düsseldorf, Germany; 2 Department of Neurology with Institute of Translational Neurology, University of Münster, 48149 Münster, Germany; 3 Laboratory of Ultrastructure, Aggeu Magalhães Institute (IAM), Recife, PE Brazil; 4 Postgraduate Program in Biosciences and Biotechnology for Health (PPGBBS), Oswaldo Cruz Foundation (FIOCRUZ-PE)/Aggeu Magalhães Institute (IAM), Recife, PE Brazil; 5 Institute for Transplantation Diagnostics and Cell Therapeutics, Medical Faculty, Heinrich-Heine-University, Düsseldorf, Germany; 6 Institute of Physiology I, University of Münster, 48149 Münster, Germany

**Keywords:** Cladribine, Cortical grey matter, Focal experimental autoimmune encephalomyelitis, Multiple sclerosis, Inflammation, Neuroaxonal damage, Neuroprotection, White matter, Electrophysiology

## Abstract

**Background:**

Cladribine is a synthetic purine analogue that interferes with DNA synthesis and repair next to disrupting cellular proliferation in actively dividing lymphocytes. The compound is approved for the treatment of multiple sclerosis (MS). Cladribine can cross the blood–brain barrier, suggesting a potential effect on central nervous system (CNS) resident cells. Here, we explored compartment-specific immunosuppressive as well as potential direct neuroprotective effects of oral cladribine treatment in experimental autoimmune encephalomyelitis (EAE) mice.

**Methods:**

In the current study, we compare immune cell frequencies and phenotypes in the periphery and CNS of EAE mice with distinct grey and white matter lesions (combined active and focal EAE) either orally treated with cladribine or vehicle, using flow cytometry. To evaluate potential direct neuroprotective effects, we assessed the integrity of the primary auditory cortex neuronal network by studying neuronal activity and spontaneous synaptic activity with electrophysiological techniques ex vivo.

**Results:**

Oral cladribine treatment significantly attenuated clinical deficits in EAE mice. Ex vivo flow cytometry showed that cladribine administration led to peripheral immune cell depletion in a compartment-specific manner and reduced immune cell infiltration into the CNS. Histological evaluations revealed no significant differences for inflammatory lesion load following cladribine treatment compared to vehicle control. Single cell electrophysiology in acute brain slices was performed and showed an impact of cladribine treatment on intrinsic cellular firing patterns and spontaneous synaptic transmission in neurons of the primary auditory cortex. Here, cladribine administration in vivo partially restored cortical neuronal network function, reducing action potential firing. Both, the effect on immune cells and neuronal activity were transient.

**Conclusions:**

Our results indicate that cladribine exerts a neuroprotective effect after crossing the blood–brain barrier independently of its peripheral immunosuppressant action.

**Supplementary Information:**

The online version contains supplementary material available at 10.1186/s12974-022-02588-7.

## Background

MS is the most common inflammatory demyelinating disorder of the CNS, characterized by inflammation and demyelination in white and grey matter regions. Since inflammatory events are central to MS disease development, current treatment approaches particularly act by restoration of a dysregulated immune response. However, neuroprotection is highly desirable in MS as in classical neurodegenerative disorders [1].

Initially established for treatment of hematologic malignancies such as hairy cell leukemia, histiocytosis or acute myeloid leukemia, cladribine was first approved for the treatment of active relapsing (RMS) and secondary progressive MS in 2017, after having shown clinical efficacy in placebo-controlled randomized clinical trials [2, 3].

Cladribine is a synthetic purine analogue prodrug, responsible for the toxic accumulation of intracellular chloro-deoxyadenosine triphosphate, resulting in sustained reduction of immune cells in the periphery [4]. Immunophenotyping studies showed that cladribine particularly affects circulating lymphocytes, e.g., CD8^+^ T cells, which are key cells underlying MS pathogenesis [5]. The high biological activity of cladribine in lymphocytes is conditioned by a high intracellular ratio of deoxycytidine kinases (DCK) to 5'-nucleotidases [4, 6]. DCK is critically involved in the metabolism of cladribine by mediating phosphorylation and promoting its accumulation in the cell. The current consensus on the selective cytotoxic effect of cladribine is centered on the expression of this enzyme [7]. By interfering with DNA synthesis and repair through incorporation into the DNA, cladribine-phosphates lead to DNA strand breaks and ultimately to cell death [4]. As small molecule drug, cladribine can further cross the blood–brain barrier and penetrate the CNS at therapeutically relevant doses [8]. Herein, cladribine is able to target dividing and non-dividing lymphocytes within the brain, and as such has been shown to reduce or eliminate intrathecal immunoglobulin synthesis in MS [9, 10]. In addition, treatment with cladribine in patients with RMS is associated with a more pronounced preservation of brain tissue than would be expected from its effect on the immune system alone, raising the hypothesis that this drug also exhibits direct neuroprotective effects [11]. Indeed, data from the literature indicate that cladribine is widely active on CNS resident cells [12–14]. In this context, cladribine has been described to modify functional properties of activated microglia [14]. In vitro stimulation of these cells with cladribine did not affect cell viability, while it amplified the gene expression of both anti- and pro-inflammatory molecules [14]. Accordingly, cladribine did not affect the apoptosis rate in a human neuronal cell line [12]. Intrathecally administrated cladribine was further able to reverse EAE-associated synaptic alterations, also pointing towards a direct neuroprotective effect of this drug in MS [13]. Of note, synaptopathy associated with CNS inflammation is a well-known phenomenon described in both RMS patients and EAE [15–17]. However, whether this beneficial and potentially neuroprotective effect of cladribine also applies to oral treatment (as currently used in practice) has not yet been clarified.

Thus, in the present investigation we explored the effects of oral cladribine treatment on the clinical EAE course, on compartment-specific immune cell alterations and integrity of the primary auditory cortex, including synaptic abnormalities associated with EAE.

While EAE is the most frequently used active immunization model of MS, inflammatory lesions following injection of myelin oligodendrocyte glycoprotein 35–55 (MOG_35–55_) peptide [15, 18] are typically confined to the spinal cord and occur in the brain in randomly distributed locations [19]. To study the neurodegenerative aspects in a reproducible localization of brain lesions, we induced focal grey matter (GM) lesions in MOG_35–55_ immunized mice by stereotactic injection of interferon gamma (IFN-γ) and tumor necrosis factor alpha (TNF-α).

Our results provide evidence that oral cladribine induces a compartment-specific immune profile in EAE mice, particular targeting circulating and brain-infiltrating leukocytes, yet with little effect on lymphoid organs. Oral cladribine, reduced the clinical deficits of EAE mice and reversed EAE-induced enhancement of the number of excitatory postsynaptic currents (EPSCs), a neurophysiological measure of glutamatergic synaptopathy associated with CNS inflammation.

## Methods

### Animals

Female, 8- to 12-week-old C57BL/6J mice were purchased from Envigo (Indianapolis, IN, USA). Mice were kept in individually ventilated cages under specific pathogen-free conditions and fed ad libitum. All animal studies were approved by institutional care committee and state committees for animal welfare (84-02.04.2015.A585). Animal experiments were conducted in accordance with the European Union normative for care and use of experimental animals and the German Animal Protection Law.

### Combined active and focal experimental autoimmune encephalomyelitis model

Induction of EAE was performed in 8- to 12-week-old female C57BL/6J mice as previously described [15]. Briefly, 10 days prior to focal EAE induction, mice were subcutaneously immunized with 200 µg of murine MOG_35–55_ peptide (Charité, Berlin, Germany) dissolved in phosphate-buffered saline (PBS, 2 mg/ml) and homogenized with complete Freund’s Adjuvant (CFA, 2 mg/ml; Merck, Darmstadt, Germany) in a 1:1 ratio. 100 μl of the resulting MOG_35–55_ emulsion was injected in each flank of anesthetized mice (isoflurane). Injection of pertussis toxin (200 ng in 100 µl PBS; Enzo Life Sciences, Farmingdale, NY, USA) was performed on day 0 and day 2 after MOG_35–55_ immunization, intraperitoneally.

**Fig. 1:**
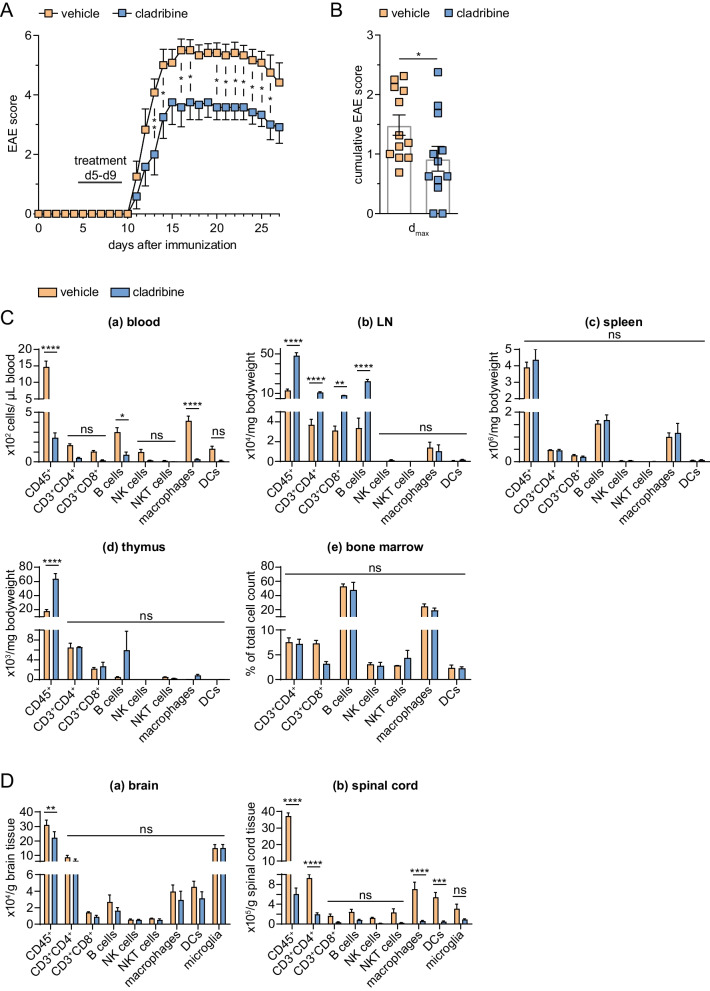
In vivo effects of cladribine on EAE score. **A** Oral treatment with cladribine (for 5 consecutive days—day 5 to 9, as indicated in the figure) induced a less severe disease course in EAE mice compared to EAE mice receiving vehicle. Statistical differences were observed in terms of development of neurological signs over the whole observation period until day 27 (two-way ANOVA, *F* (1, 22) = 9.446, *p* = 0.0056, *n* = 12 for each group). **B** The bar graph depicts the cumulative EAE score of both experimental groups on the day of maximal clinical deterioration (*d*_max_*, *unpaired Mann–Whitney *U* test, *U* = 34.50, *p* = 0.0295, *n* = 12 for each group). **C** Immunophenotypings of the peripheral blood (a), LNs (b), spleen (c), thymus (d) and bone marrow (e) at *d*_max_ were performed by flow cytometry. Immune cell profiles of mice treated with cladribine were compared to those receiving vehicle. Statistically significant differences were obtained by performing two-way ANOVAs complemented by Bonferroni test for multiple comparisons, *n* = 3 for each group. *p*-values: 0.0019 (a, blood), 0.0003 (b, LN), 0.6227 (c, spleen), 0.0016 (d, thymus) and 0.3734 (e, bone marrow). **D** Flow cytometric analyses were used, to evaluate the immune cell distribution in the brain (a) and spinal cord (b) of EAE mice, either treated with cladribine or vehicle at *d*_max_. Statistically significant differences were obtained by performing two-way ANOVAs complemented by Bonferroni test for multiple comparisons, *n* = 3 for each group. *p*-value was 0.2486 for brain, < 0.0001 for spinal cord. *p* > 0.05 = ns, *p* < 0.05 = *, *p* < 0.01 = **, *p* < 0.001 = ***, *p* < 0.0001 = ****

**Fig. 2:**
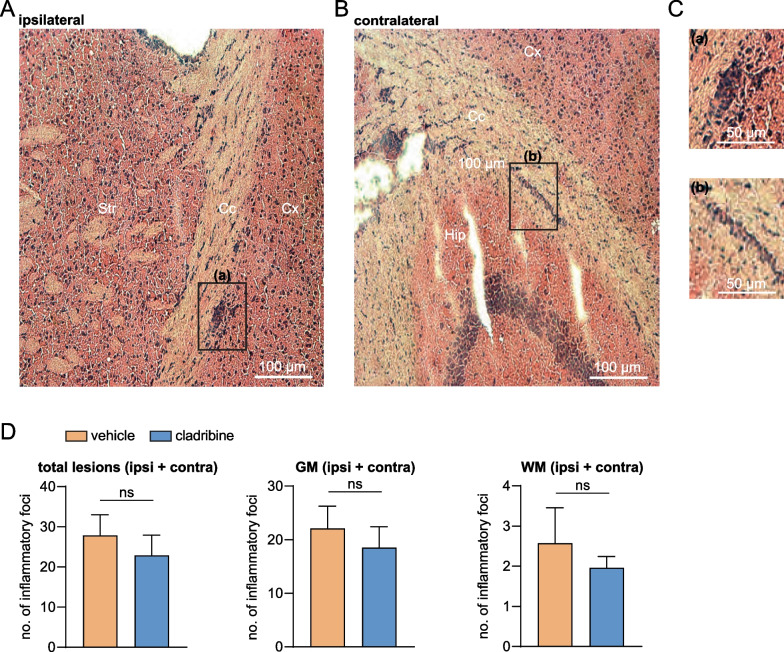
In vivo cladribine treatment does not affect the number of inflammatory foci in the brain of EAE mice. Hematoxylin and eosin (H&E) staining of coronal sections comparing inflammatory CNS lesions in vehicle- and cladribine-treated EAE mice. Images were acquired with a Zeiss Axio Scope.A1 (Zeiss, Göttingen, Germany) using tenfold objectives. Exemplary H&E stainings of the ipsilateral hemisphere (**A**) and the contralateral hemisphere (without stereotactic cytokine injection, **B**) are shown. Scale bar 100 µm. *Cc* corpus callosum, *Cx* cortex, *Hip* hippocampus, *Str* striatum. **C** Representative lesions of the ipsilateral (**a**) and the contralateral (**b**) hemisphere are magnified. Scale bar 50 μm. **D** Histograms showing the quantification of inflammatory lesions in both hemispheres in coronal sections of vehicle- and cladribine-treated EAE mice. Lesions are classified topologically (grey (GM) and white (WM) matter). No statistic difference was obtained following unpaired Mann–Whitney* U* test (*p* > 0.05). *n* = 3, 35 slices per animal were analyzed and the mean per slice and mouse was used for statistical analysis

**Fig. 3:**
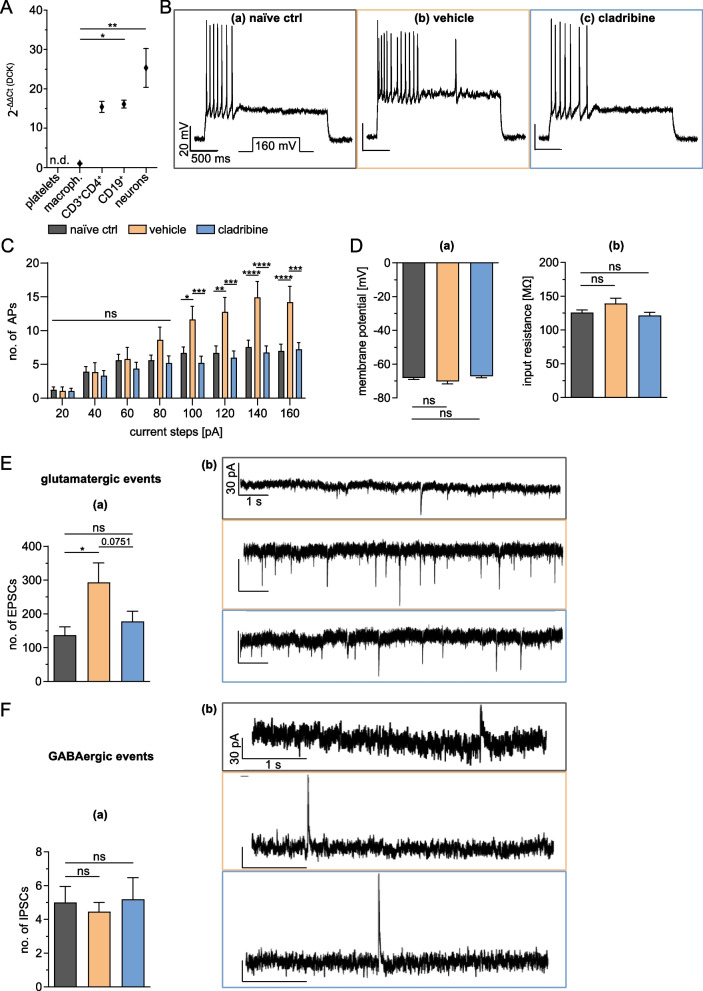
The capacity of cladribine to affect neurons of the primary auditory cortex. **A** mRNA expression of deoxycytidine kinase (DCK) was quantified by quantitative polymerase chain reaction (qPCR) in magnetic-associated cell-sorted (MACS) neurons, compared to macrophages (macroph.; CD45^high^CD11b^high^), CD3^+^CD4^+^ and CD19^+^ lymphocytes. Platelets served as negative control. Results were normalized to expression levels in macrophages and are depicted as 2^−∆∆Ct^ values (*n* = 9 for macrophages, *n* = 5 for all other cell types, Kruskal–Wallis test). **B**–**F** All electrophysiological recordings were obtained at *d*_max_ by current-clamp mode (**B**–**D**) or voltage-clamp mode (**E** + **F**). Four mice were examined per experimental group. **B** Representative traces recorded at a potential of − 60 mV (set by DC current injection) in current-clamp mode from one animal. A depolarizing current step of + 160 pA triggered the generation of action potentials (APs) in all experimental groups. **C** Mean bar graph indicating the number of APs generated in response to depolarizing steps of increased intensity ranging from + 20 to + 160 pA. The number of generated APs increased with increasing depolarization under all experimental conditions. Statistically significant differences were obtained starting from + 100 pA (two-way ANOVA, *F* (2.59) = 5.061, *p* = 0.0094, *n* = 23—naïve ctrl, 14—vehicle, 25—cladribine). **D** Resting membrane potential (in mV; **a**) and input resistance (in MΩ; **b**) of neurons of the auditory cortex are depicted indicating no differences between vehicle- vs. cladribine-treated EAE mice compared to naïve controls (unpaired Mann–Whitney* U* test, *p* > 0.05, *n* = 23—naïve ctrl, 14—vehicle, 24—cladribine). **E** Effects of oral treatment with cladribine on glutamatergic transmission in voltage-clamp mode recorded as excitatory postsynaptic currents (EPSCs). Upon EAE induction, the number of EPSCs (**a**) increased significantly compared to naïve control (unpaired Mann–Whitney *U* test, *p* = 0.0401, *n* = 28—naïve ctrl, 16—vehicle). Upon cladribine treatment, a trend for a reduction in EPSCs compared to vehicle-treated mice was observed, although not reaching significance (unpaired *t* test, *p* = 0.0751, *n* = 16—vehicle, 19—cladribine). Exemplary electrophysiological traces of voltage-clamp recordings (**b**) show exemplary EPSC events in recorded neurons from naïve control, vehicle-treated and cladribine-treated EAE mice. **F** Bar graph illustrating the number of inhibitory postsynaptic currents (IPSCs; **a** indicating that neither the experimental EAE itself nor oral cladribine treatment did significantly affect the GABAergic transmission (unpaired Mann–Whitney *U* test, *p* > 0.05, *n* = 19—naïve ctrl, 11—vehicle, 16—cladribine). Representative electrophysiological traces of voltage-clamp recordings of IPSCs (**b**) from all three experimental groups. *p* > 0.05 = ns, *p* < 0.05 = *, *p* < 0.01 = **

**Fig. 4:**
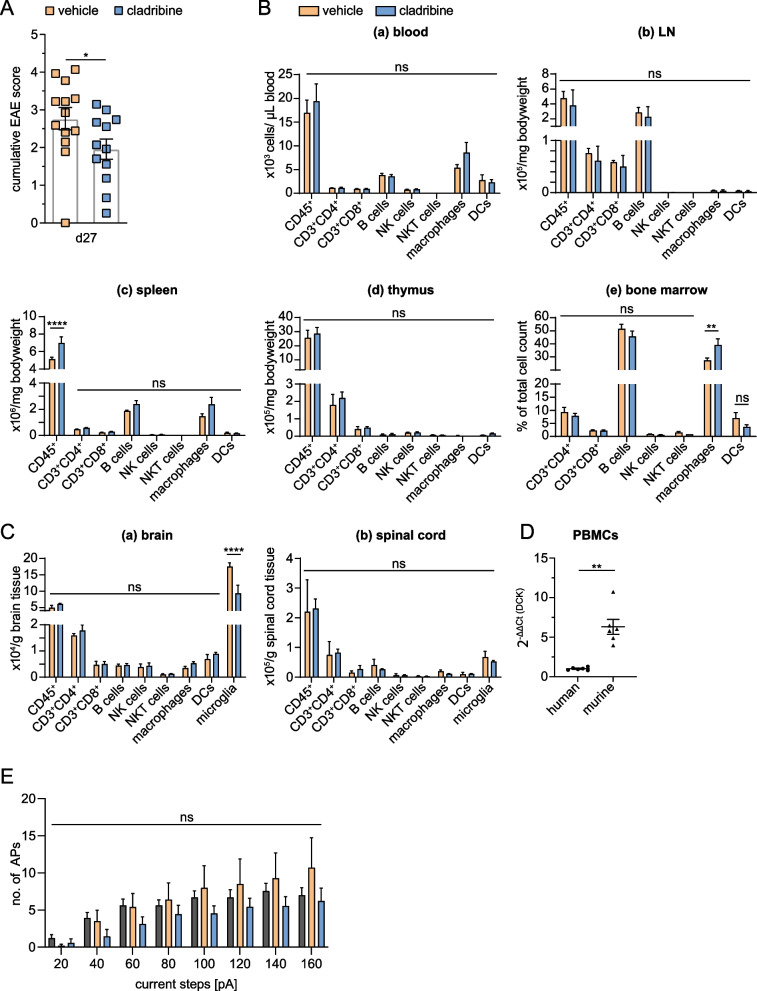
Immunosuppressive and neuroprotective effects of cladribine are transient on cellular level. **A** Oral treatment with cladribine induced a less severe disease score on day 27 in EAE mice compared to EAE mice receiving vehicle treatment (unpaired Mann–Whitney *U* test, *p* = 0.0386, cladribine-treated (*n* = 12) vs. vehicle-treated (*n* = 13)). **B** Immunophenotypings of the peripheral blood (**a**), LNs (**b**), spleen (**c**), thymus (**d**) and bone marrow (**e**) at day 27 post-EAE induction were performed by flow cytometry. Immune cell profiles of mice treated with cladribine were compared to those receiving vehicle. No statistically significant differences could be observed (two-way ANOVAs, *n* = 3 for each group. *p*-values > 0.05). **C:** Flow cytometric analysis of the immune cell distribution in the brain (**a**) and spinal cord (**b**) of EAE mice on day 27 post-immunization, either treated with cladribine or vehicle, shows no significant differences between both experimental groups (two-way ANOVAs, *n* = 3 for each group. *p*-values > 0.05). **D** mRNA expression of deoxycytidine kinase (DCK) was quantified by quantitative polymerase chain reaction (qPCR) in murine PBMCs and normalized to human PBMCs. Results are depicted as 2^−∆∆Ct^ values (*n* = 6 for both species, unpaired Mann–Whitney* U *test, *p* = 0.0022). **E** Electrophysiological recordings of APs were obtained by recording in current-clamp mode. Mean bar graph indicating the number of APs recorded on day 27 post-EAE induction in response to depolarizing steps of increased intensity ranging from + 20 to + 160 pA. No significant alterations could be observed between groups (two-way ANOVA, *n* = 23—naïve ctrl, 10—vehicle, 9—cladribine. *p*-values > 0.05). *p* > 0.05 = ns, *p* < 0.05 = *, *p* < 0.01 = **

Ten days after MOG_35–55_ immunization, mice were deeply anesthetized and mounted on a stereotactic device. Using the following coordinates (anteroposterior, − 2.18 mm; lateral, 4.2 mm from bregma; and dorsoventral, 1 mm from the brain surface for the auditory cortex), a hole was drilled through the skull. Two μl of a solution containing the proinflammatory cytokines TNF-α (150 U; Merck) and IFN-γ (800 U; Merck) dissolved in PBS were slowly injected into the left auditory cortex. The contralateral hemisphere (right side) served as control. Mice were killed at the day of maximal clinical deterioration (*d*_max_, Additional file 1: Fig. S1) for experimental setting one (Figs. 1, 2, 3) or 17 days post-injection (day 27) to determine long-term effects of cladribine treatment in a chronic EAE state (Fig. 4). In all experimental steps, mice were randomly assigned to the operators by an independent person not involved in data analysis. Surgery and evaluation of all read-out parameters were performed in a blinded manner.

### Oral cladribine treatment

Five days post-immunization, cladribine (10 mg/kg; Merck) was administered daily via oral gavage in 0.5% aqueous carboxymethylcellulose for 5 days (further referred to as ‘cladribine-treated EAE group’). The vehicle group received 0.5% aqueous carboxymethylcellulose for 5 days at the same period (further referred to as ‘vehicle-treated EAE group’). Moreover, an additional control group of naïve mice (without EAE or any oral treatment), further referred to as ‘controls’, was used for ex vivo electrophysiological analysis. Prior to the EAE experiments, titration experiments of cladribine were conducted, indicating 10 mg/kg as the appropriate dose to reach an adequate brain concentration of approximately 80–100 ng/g over a period of 2.5 h (Additional file 2: Fig. S2). This time interval was chosen in advance, as cladribine is rapidly available following oral administration and maximum plasma levels are found within 1 to 2 h of ingestion [8].

### Functional outcome tests

Health status and disease progression (weight, disease score, general appearance and performance) of mice was monitored on a daily basis by two blinded investigators. Disease severity was defined using the following scoring system: grade 0, no abnormality; grade 1, limp tail tip; grade 2, limp tail; grade 3, moderate hindlimb weakness; grade 4, complete hindlimb weakness; grade 5, mild paraparesis; grade 6, paraparesis; grade 7, heavy paraparesis or paraplegia; grade 8, tetraparesis; grade 9, quadriplegia or premoribund state; or grade 10, death. Animals presenting with a score ≥ 7 for more than three consecutive days or showing a score of 8 (independent of the time period) were killed and the last score observed was included in the analysis until the end of the experiment, respectively. Mice showing a bodyweight reduction of more than 20% compared to their starting bodyweight (bw) were excluded from experiments as well. The cumulative disease score was calculated as the sum of the daily clinical scores of each mouse during the EAE observation period and reported as an average within each group (mean ± SEM). dmax refers to the day of maximum clinical deterioration representing the day on which the majority of mice in the experiment had the highest clinical score (i.e., day with highest cumulative disease score). We determined dmax based on our long-term experience with the EAE model [15, 18, 20–24]. Using our standardized EAE protocol, we observed a typical chronic course over 25–30 days (EAE duration depending on experimental setup) with onset of clinical symptoms about day 10 and disease maximum (dmax) about day 15–18 post-immunization followed by a remission of symptoms [18, 20]. In the current study, dmax was reached on day 17 and maximum EAE duration was set to 27 days.

### Tissue preparation

At *d*_max_ and day 27 post-EAE induction, blood was taken by heart puncture under deep isoflurane anesthesia. Afterwards, mice were perfused via the left ventricle with PBS. Directly after cardiac perfusion, brains, spinal cords, lymph nodes (LN), spleens, thymi and femur bones were removed and single-cell suspensions were prepared.

Blood was treated with erythrocyte lysis buffer (150 mM NH4Cl, 10 mM KHCO3, 0.1 mM EDTA; pH 7.3). Organs (mouse spleen, LN, thymus and bone marrow) were homogenized by a 40-μm cell strainer (BD Biosciences, Germany). Homogenates were rinsed with washing medium (Dulbecco’s modified Eagle’s medium, DMEM; Invitrogen, Waltham, MA, USA) containing 1% fetal bovine serum (FBS, ScienCell, Carlsbad, CA, USA) and 1% antibiotics (penicillin/streptomycin, Sigma-Aldrich, St. Louis, MO, USA). Erythrocytes in the splenocyte suspension were lysed with erythrocyte lysis buffer for 30 s. Then, lysis was stopped by addition of washing medium. Single cell suspensions were washed once again and resuspended in the desired buffer for subsequent applications.

For purification of CNS-infiltrating leukocytes, brain and spinal cord tissues were cut into pieces, and mechanically homogenized in HBSS by an insulin syringe. After a 30-min enzymatic digestion with a collagenases, DNase and trypsin-inhibitor mix, suspension was layered on a density gradient using Lymphoprep™ (Fresenius, Germany), and separated by centrifugation (18 min at 790*g* without acceleration and break). After isolation, the pellet was washed and resuspended in the respective staining buffer. To quantify numbers of cells isolated from the CNS, beads (Beckman Coulter, Brea, CA, USA) were added.

### Flow cytometry

Phenotyping of different immune cell subsets was performed using flow cytometry. Therefore, immune cells isolated from blood, spleen, thymus, LN, bone marrow and CNS (brain and spinal cord) were characterized by staining for CD3, CD4, CD8, CD11a/b, CD11c, CD25, CD44, CD45, CD45R/B220, CD49d, CD62L, CD69 and NK1.1 (for details see Table 1 and Additional file 1: Fig. S3). Briefly, single-cell suspensions were stained for 30 min at 4 °C with the appropriate combination of indicated fluorescence-labeled monoclonal antibodies in PBS, containing 2 mM EDTA and 0.1% bovine serum albumin (Sigma-Aldrich). Corresponding isotype controls were used for all stainings. For blocking of Fc receptors, cells were preincubated with purified anti-CD16/CD32 antibody for 10 min on ice prior to immunostaining. Fixable viability dye eFluor (Thermo scientific, Waltham, MA, USA) was used for live/dead staining. Concentrations of antibodies were carefully titrated prior to experiments. Flow cytometric analysis of stained cells was performed following standard protocols. Cells were analyzed on a Gallios Flow Cytometer (Beckman Coulter) and a CytoFLEX S (Beckman Coulter) using *Kaluza Analysis Software* (Beckman Coulter).

**Table 1:** Antibodies used for flow cytometry

Antigen	Reactivity	Supplier	Order no.
*Flow cytometry*			
CD3	Mouse	BioLegend	100218
CD4	Mouse	BioLegend	100531
CD8a	Mouse	BioLegend	100730
CD11a	Mouse	BioLegend	153103
CD11b	Mouse/human	BioLegend	101263
CD11c	Mouse	BioLegend	117306
CD25	Mouse	BioLegend	101915
CD44	Mouse	BioLegend	103049
CD45	Mouse	BioLegend	103116
CD45R/B220	Mouse	BioLegend	103211
CD49d	Mouse	BioLegend	103627
CD62L	Mouse	BioLegend	104437
CD69	Mouse	BioLegend	104521
NK1.1	Mouse	BioLegend	108710

### Isolation of murine peripheral blood mononuclear cells (PBMCs)

Female mice (6 replicates, 3 mice per replicate) were anesthetized with isoflurane. Blood was withdrawn directly from the heart using a 1-ml syringe filled with 50 µl of EDTA (2 mM). Blood from 3 mice was pooled in one 2-ml tube and transferred to a 50-ml conical tube. The remaining blood in the 2-ml tubes was washed out with 2 mL D-PBS + 2% FCS. 5 ml of D-PBS + 2% FCS were added to the blood/D-PBS mixture to obtain a final volume of 13 ml. Next, the blood was layered in SepMate™-50 IVD tubes (STEMCELL Technologies, France) pre-filled with Cytiva Ficoll-Paque™ PLUS (density 1.077 ± 0.001 g/ml; Thermo scientific). Samples were centrifuged for 20 min at 12,000 rpm at room temperature. The upper phase with PMBCs was transferred to a new 50-ml conical tube. Samples were filled with wash medium (DMEM supplemented with 1% FCS, 1% l-glutamine and 1% penicillin/streptomycin) to a total volume of 50 ml. Subsequently, centrifugation for 10 min at 300*g* and 4 °C was performed. The supernatant was discarded, and the pellet was resuspended in DMEM for cell counting using a Neubauer chamber. We acquired approximately 5 × 10^6^ PBMCs per replicate.

### Isolation of human PBMCs

Human PBMCs were isolated from 6 healthy donors. Blood samples of control subjects without an autoimmune or neuroinflammatory disorders (*n* = 6) were included in this study. All cases presented with non-specific complaints and underwent blood sampling during a routine diagnostic examination conducted to rule out any neurological condition. None of the healthy controls suffered from a neurological disorder, nor did they show any specific abnormalities during the neurological examination. All patients included in this study gave their written informed consent in accordance with the Declaration of Helsinki and a protocol approved by the Ethics Committee of the University of Duesseldorf (5951R). 

On the day of experiment, blood was drawn from healthy donors, collected in 10-ml EDTA tubes (BD Diagnostics Systems, Franklin Lakes, NJ, USA), and diluted with PBS in a 1:1 ratio. Isolation was performed using Cytiva Ficoll-Paque™ PLUS (density 1.077 ± 0.001 g/ml; Thermo scientific) within a SepMate™-50 IVD tube (STEMCELL Technologies) according to the manufacturer’s instructions. Cells were counted and cryopreserved at approximately 1 × 10^7^ cells per 1 ml.

### Fluorescence-activated cell sorting (FACS) of macrophages

First, CD11b^+^ cells were isolated from murine adult brain and spinal cord as previously described [25]. Nine biological replicates were collected. For each biological replicate, 3 naïve C57BL/6J mice (female, 15–25 weeks old, 18–35 g bw) were pooled. Second, CD11b^+^ cells were stained with FITC anti-mouse/human CD11b antibody (clone M1/70; BioLegend, San Diego. CA, USA) 1:50 and APC/Cyanine7 anti-mouse CD45 antibody (clone 30-F11; BioLegend) 1:200 in PBS for 15 min at room temperature. Concentrations of antibodies were carefully titrated prior to experiments. FACS staining was performed following standard protocols. Afterwards, macrophages were analyzed and sorted by use of a MoFlo XDP, Cell Sorter (Beckman Coulter; 100 m$\upmu$ nozzle, pressure: 26 psi) using Summit Analysis Software version 5.4.0 (Beckman Coulter). Macrophages were identified as CD45^high^CD11b^high^, while microglia were suspected to be CD45^int^CD11b^high^ [25] (for gating strategy see Additional file 4: Fig. S4).

### RNA isolation and real-time quantitative PCR (qPCR)

CD3^+^CD4^+^ and CD19^+^ lymphocytes were isolated via magnetic-activated cell sorting (MACS) from cervical LN of adult naïve mice according to the manufacturer’s instructions (Miltenyi Biotec, Germany). Adult neurons were isolated as previously described [25]. Subsequently, the expression of DCK was quantified by qPCR. Platelets were isolated as previously described and served as negative control, as they have no nucleus and showed no DCK expression [26].

RNA was isolated with the Quick-RNA Microprep Kit (Zymo Research) following the manufacturer’s protocol. Tissue homogenates and cells were lysed in 300 μl RNA lysis buffer, followed by sample clearing. The supernatant was mixed with 95–100% ethanol and transferred to the column. In-column DNAse treatment was performed. After washing and drying the column, RNA was eluted by pre-warmed DNase/RNase-free water (15 μl). RNA quality was measured with Nanodrop by A260/A280 and A260/A230 ratios.

Reverse transcription was performed with Maxima Reverse Transcriptase (Thermo scientific) and random hexamer primers. One-hundred ng cDNA was used for real-time qPCR with TaqMan Master Mix (Maxima probe/ROX, Applied Biosystem). To this end, 1 μM of each primer (target primers: murine DCK, Mm00432794_m1; human DCK, #4331182; Thermo scientific) or 1 μM house-keeping primer for the respective control (18 s, #4333760 T), 10 μl of maxima probe/carboxyrhodamine (ROX) fluorescent dye, 4 µl of DNA-free aqua and 100 ng cDNA (4 µl) were mixed. Run was performed on a StepOnePlus™ Real-Time PCR System (Applied Biosystems) according to the following steps: hold—2 min 50 °C, initial denaturation—10 min 95 °C, amplification—(40x) 10 s 95 °C—45 s 58 °C—1 min 72 °C. Data were analyzed with the StepOne software (Applied Biosystems, v2.1) calculating 2^−∆∆Ct^ values (the ratio of DCK expression to the house-keeping gene, normalized to its expression in macrophages or human PBMCs, respectively).

### Histology—hematoxylin–eosin (H&E) staining

In order to verify the injection site, and to quantify the amount of white and grey matter lesions in the cladribine-treated EAE compared to the vehicle-treated EAE group, brains were used for histopathological evaluation. Mice were perfused through the left ventricle with PBS for 5 min under deep isoflurane anesthesia. Brains were removed, and immediately frozen in embedding medium (Tissue-Tek® O.C.T.™ compound, Sakura Finetek, Germany). Cryopreserved brain slices (10 µm thick) were stained with hematoxylin and eosin (H&E) using standard protocols. Light microscopy and AxioVision software were used to determine the number of inflammatory lesions per animal. Lesions were classified by anatomy (grey and white matter) and location (ipsilateral (cytokine injection side) and contralateral hemisphere) for both groups. Thirty-five slices per mice were analyzed, three mice per experimental group. Means ± SEM (standard error of the mean) per slice and mouse were calculated and used for further statistical analyses.

### Electrophysiological experiments

In order to investigate neuronal excitability, we analyzed the firing pattern of auditory pyramidal cortical neurons (layer 4) in current-clamp mode. In detail, at *d*_max_ and day 27 (for long-term studies, Additional file 1: Fig. S1), the animals were deeply anesthetized, and the brains quickly removed. Brains were glued onto a cutting plate with the help of an agar block to cut (vibratome from Leica) coronal slices (250–300 µm of thickness) containing preserved network structure of the auditory cortical network [27]. Cutting was performed in ice-cold artificial cerebrospinal fluid (ACSF) solution (200 mM sucrose, 10 mM glucose, 20 mM PIPES, 2.5 mM KCl, 10 mM MgSO_4_, 0.5 mM CaCl_2_; pH 7.35).

Electrical recordings were performed at room temperature in carbonated ACSF as extracellular solution containing the following (in mM): NaCl, 125; KCl, 2.5; NaH_2_PO4, 1.25; HEPES, 30; MgSO_4_, 2; CaCl_2_, 2; glucose, 10; pH 7.35 with NaOH; 305 mOsm/kg in a submerged chamber on an upright microscope (Zeiss, Germany). Intracellular recordings were performed in visually identified neurons of the layer 4 of the primary auditory cortex, using a ZEN 2.5 camera (Zeiss) and were governed by using the PatchMaster software (HEKA, Germany).

Recording pipettes were advanced towards individual neurons in the slice under positive pressure and visual control. The membrane patch was then ruptured by suction and the membrane potential was monitored using a double patch amplifier (HEKA EPC 10). Whole-cell patch clamp recordings were made with borosilicate glass pipettes (GC150TF-10, Harvard Bioscience, Holliston, MA, USA) for both voltage- and current-clamp mode.

Neurons were challenged with the administration of a series of depolarizing current steps of increasing intensity leading to the generation of action potentials (APs). A total of 8 current steps of increasing intensity (from + 20 to + 160 pA, 2.5 s duration) were applied and the number of APs was taken as read-out. APs were counted semi-automatically using the software PEAK (Meuth IT Consulting) and confirmed by visual inspection of each recorded step. To investigate changes in cellular excitability threshold and the number of fired APs for the same baseline, recordings were performed at a holding potential of − 60 mV that was set by DC current injection.

To study spontaneous postsynaptic currents, including EPSCs or inhibitory postsynaptic currents (IPSCs), the recording pipettes were filled with an internal solution of the following composition (in mM): NaCl, 10; K-gluconate, 88; K3-citrate, 20; HEPES, 10; BAPTA, 3; phosphocreatine, 15; MgCl2, 1; CaCl2, 0.5; Mg-ATP, 3; Na-GTP, 0.5; set to pH 7.25 with KOH and osmolality of 295 mOsmol/kg. To record both, spontaneous glutamatergic- and GABAergic currents in the same experiment, cells were clamped to a holding potential of − 60 mV (glutamatergic currents), and subsequently to 0 mV to record GABAergic currents.

Spontaneous synaptic events were acquired using PatchMaster (HEKA) and analyzed using MiniAnalysis software 6.0. (Synaptosoft Inc., Fort Lee, NJ, USA). The detection threshold of EPSCs was set at twice the baseline noise and once the baseline for IPSCs. With this recording setting, all technically detected positive spikes represented GABAergic currents (IPSCs) and negative spikes constituted glutamatergic currents (EPSCs). Counts were confirmed for each cell and experiment by visual inspection of each recorded trace.

### Cell isolation from spleen and LN

Spleens or LNs were homogenized by a 40-µm cell strainer and washed with 10 ml washing medium (DMEM, 1% FCS, 1% penicillin/streptomycin). Erythrocytes in the cell suspension were lysed with ACK buffer (150 mM NH4Cl, 10 mM KHCO3, 0.1 mM EDTA, pH 7.3) for 30 s, stopped by addition of washing medium. Single cell suspensions were washed once again and resuspended in splenocyte complete medium (DMEM, 10 mM HEPES, 25 μg/ml gentamicin, 50 μM mercaptoethanol, 5% fetal calf serum, 2 mM glutamine, and 1% non-essential amino acids (Cambrex, Verviers, Belgium)).

### Leukocyte organ culture

Murine cervical LNs and small pieces of spleen were placed on 24-well plate Costar Transwell inserts with a 3.0 µm pore polyester membrane (Corning, Lowell, MA, USA). 600 µl organ culture medium (RPMI, 10% FCS, 1% penicillin/streptomycin) were placed in the lower chamber and 100 µl in upper chamber. The organs were treated either with 0.1 µM cladribine or with the responding vehicle in the control group. After 7 days, all migrated cells were collected and analyzed by flow cytometry. The organs were homogenized according to the protocol described for splenocyte isolation. Cells were analyzed by flow cytometry using the following markers: CD4, CD8, CD45R/B220, CD11a, CD25, CD44, CD49d, CD62L, CD69 (for details see Table 1 and “Flow cytometry” section).

### Proliferation assay

Lymphocytes were isolated according to the protocol described above and labeled with VybrantTM CFDA SE Cell Tracer (12.5 µM) in 2 ml PBS + 2% FCS for 10 min at 37 °C, followed by addition of 10 ml cold washing buffer and incubation on ice for 10 min. After washing the cells, they were seeded into 96-well plates (U-bottom) coated with 1 µg/ml anti-CD3. Soluble anti-CD28 (2 µg/ml) was added to the splenocyte complete medium as indicated in the respective experiments. After plating the cells, the different groups were treated with either 0.1 µM cladribine, 1.0 µM cladribine or the vehicle, respectively. Cells were cultured for 3 days (37 °C, 5% CO_2_) prior to flow cytometry analysis. Cells were analyzed by flow cytometry using the following markers: CD4, CD8, CD45R/B220, CD11a, CD25, CD44, CD49d, CD62L, CD69 (for details see Table 1 and “Flow cytometry” section).

### Statistical analysis

Results are displayed as means ± SEM unless indicated otherwise. For column-based data, Gaussian distribution was evaluated by D’Agostino–Pearson normality test. Dependent on normality for analysis of two groups, two-tailed *t* test (unpaired/paired) or Mann–Whitney *U* test was used as appropriate. If more groups were compared, we applied one-way ANOVA, complemented by Bonferroni test for multiple comparisons for parametric data, or the Kruskal–Wallis test including Dunn’s post-test for non-parametric data. Comparison of EAE data was performed using two-way ANOVA.

The level of significance was labeled according to the *p*-values: *p* values > 0.05 were classified as not significant, *p* < 0.05 (*) as significant, *p* < 0.01 (**), *p* < 0.001 (***) and *p* < 0.0001 (****) as highly significant. Analyses and graphs were prepared using Prism 9.1.2 (Graph Pad, San Diego, CA, USA).

### Data availability statement

The data that support the findings of this study are available from the corresponding author upon reasonable request.

## Results

### In vivo effect of cladribine on clinical score and immune cell composition in a mouse model of autoimmune neuroinflammation

We used the combined active and focal EAE as a paradigmatic model of autoimmune neuroinflammation to analyze the consequences of oral cladribine treatment on disease course and corresponding immune profiles in mice. To this end, C57BL/6 J mice were immunized with MOG_35–55_ peptide 10 days prior to stereotactical injection of proinflammatory cytokines (IFN-γ and TNF-α) in the auditory cortex to induce cortical grey and white matter lesions, respectively. Oral cladribine treatment for 5 days led to a significantly reduced disease severity throughout the whole observation period compared to the vehicle-treated group (Fig. 1A). In line with a recently published study of intrathecal cladribine administration [13], animals orally treated with cladribine showed only mild clinical symptoms during the whole observation period (exemplary: cladribine- vs. vehicle-treated EAE at *d*_max_: 0.92 ± 0.21 vs. 1.48 ± 0.17; *p* < 0.05; unpaired Mann–Whitney* U* test, *n* = 12 animals for each group; Fig. 1B).

Flow cytometric analysis of peripheral blood indicated a significant reduction of leukocyte (subsets) in orally treated mice (*n* = 3) compared to those treated with vehicle (*n* = 3) at *d*_max_ (cladribine- vs. vehicle-treated EAE, leukocytes: 2.42 ± 0.50 vs. 14.70 ± 1.76 * 10^2^ cells/µl, *p* < 0.001; and B cells: 0.72 ± 0.28 vs. 3.00 ± 0.46 * 10^2^ cells/µl, *p* < 0.05, two-way ANOVA, complemented by Bonferroni test for multiple comparisons; Fig. 1C). Of note, cladribine treatment also reduced innate immune cell numbers in the peripheral blood of EAE mice (macrophages: 0.24 ± 0.07 vs. 4.15 ± 0.47 * 10^2^ cells/µl, *p* < 0.0001), although those cells remained within the lower limit of normal or were largely unaffected in human studies [28–30]. T cell subsets, NK cells and natural killer T cells (NKT) showed a trend for reduction, although not significant (CD3^+^CD4^+^ T cells: 0.39 ± 0.05 vs. 1.67 ± 0.15 * 10^2^ cells/µl; CD3^+^CD8^+^ T cells: 0.16 ± 0.03 vs. 1.03 ± 0.12 * 10^2^ cells/µl; NK cells: 0.15 ± 0.03 vs. 1.00 ± 0.25 * 10^2^ cells/µl; NKT cells: 0.02 ± 0.01 vs. 0.11 ± 0.03 * 10^2^ cells/µl; for all cell subsets *p* > 0.05).

In contrast to the immune cell profile of the peripheral blood, cladribine treatment did not affect immune cell composition of the spleen and bone marrow (Fig. 1C). Moreover, it showed significantly paradoxical findings in the LN and thymus of EAE mice treated with cladribine in terms of leukocyte levels (cladribine- vs. vehicle-treated EAE, LN—leukocytes: 48.37 ± 2.88 vs. 13.17 ± 1.42 * 10^4^/mg bodyweight (bw), *p* < 0.0001; thymus—leukocytes: 63.80 ± 7.18 vs. 17.70 ± 2.54 * 10^3^/mg bw, *p* < 0.0001) next to CD3^+^CD4^+^, CD3^+^CD8^+^ T cells and B cell numbers (LN only; CD3^+^CD4^+^ T cells: 11.20 ± 0.82 vs. 3.70 ± 0.55 *10^4^/mg bw, *p* < 0.0001; CD3^+^CD8^+^ T cells 8.17 ± 0.13 vs. 3.10 ± 0.47 *10^4^/mg bw, *p* < 0.01; B cells: 22.43 ± 1.74 vs. 3.37 ± 1.01 *10^4^/mg bw, *p* < 0.0001; Fig. 1C).

Next, we analyzed whether peripheral immune cell alterations were accompanied with reduced immune cell infiltration into the brain parenchyma compared to vehicle-treated animals (*n* = 3 in each group).

Cladribine treatment resulted in a notably lower number of leukocytes compared to the vehicle-treated group (cladribine- vs. vehicle-treated EAE, brain: 22.20 ± 4.18 vs. 31.00 ± 3.45 * 10^4^/g brain tissue, *p* < 0.01; spinal cord: 6.03 ± 1.21 vs. 37.27 ± 1.93 * 10^5^/g spinal cord tissue, *p* < 0.0001; two-way ANOVA, complemented by Bonferroni test for multiple comparisons, Fig. 1D).

Detailed analysis of leukocyte subset distribution revealed that cladribine-treated mice display reduced numbers of T cells (CD3^+^CD4^+^: 1.95 ± 0.33 vs. 9.23 ± 0.75 *10^5^/g spinal cord tissue, *p* < 0.0001) as well as macrophages and dendritic cells (macrophages: 0.56 ± 0.02 vs. 7.05 ± 1.37 * 10^5^/g spinal cord tissue, *p* < 0.0001; dendritic cells: 0.45 ± 0.13 vs. 5.40 ± 0.98 * 10^5^/g spinal cord tissue, *p* < 0.001) in the spinal cord; however, they did not show alterations in the levels of CD3^+^CD8^+^ T cells, B cells, NK cells, NKT cells and microglia compared to vehicle-treated mice.

In summary, cladribine treatment ameliorated autoimmune neuroinflammation in vivo. Our findings pointed to a yet unknown compartment-specific effect of oral cladribine treatment in mice.

### Cladribine shows a tendency to reduce the incidence of inflammatory brain lesions in comparison to vehicle-treated controls.

In order to evaluate whether the aforementioned immune cell alterations provide a pathophysiological relevance during the EAE course, we performed histological studies to characterize inflammatory brain lesions in both groups (*n* = 3 in each group, Fig. 2A–C). Our combined active and focal EAE model allowed us to study both cortical grey and subcortical white matter inflammatory lesions, respectively.

In both experimental groups—albeit not significant—we appreciated that the ipsilateral hemisphere (site of stereotactic cytokine injection) showed more inflammatory lesions compared to the contralateral one (cladribine-treated EAE: ipsilateral 13.09 ± 1.88 vs. contralateral 7.95 ± 1.90, *p* = 0.1399; vehicle-treated EAE: ipsilateral 14.84 ± 3.56 vs. contralateral 9.87 ± 1.79, *p* = 0.2046; paired *t* tests, data not shown). GM lesions exceeded white matter (WM) lesions significantly (cladribine-treated EAE: GM 18.51 ± 3.93 vs. WM 1.96 ± 0.28, *p* = 0.0460; vehicle-treated EAE: GM 22.13 ± 4.15 vs. WM 2.57 ± 0.89, *p* = 0.0283; paired *t* tests, Fig. 2D). Of note, cladribine treatment showed a trend to reduce both, WM and GM lesions, although not reaching significance (cladribine-treated vs. vehicle-treated EAE, total lesions: 22.87 ± 5.06 vs. 27.84 ± 5.15; GM: 18.51 ± 3.93 vs. 22.13 ± 4.15; WM: 1.96 ± 0.28 vs. 2.57 ± 0.89, *p* > 0.05 in each comparison, unpaired Mann–Whitney *U* test, Fig. 2D).

These data suggest that cladribine-induced leukocyte depletion did not result in a significant reduction of inflammatory lesions in the brain, indicating undergoing neuroinflammation.

### Ex vivo consequences of cladribine treatment on neuronal network functions

Ruggieri et al*.* [12] recently showed that neuronal apoptosis was not affected by cladribine treatment in vitro. However, they did not analyze the susceptibility of neurons to cladribine conditioned by their expression of DCK. Therefore, we first explored the expression levels of DCK in neurons, prior to study potential neuroprotective effects of cladribine on these cells. Interestingly, in our study, neurons showed comparable mRNA expression levels of DCK, as compared to T- and B-lymphocytes (neurons: 23.35 ± 4.95 2^−∆∆Ct^ value; CD3^+^CD4^+^ T cells: 15.44 ± 1.42 2^−∆∆Ct^ value; CD19^+^ B cells: 16.13 ± 0.98 2^−∆∆Ct^ value; *p* > 0.05, Kruskal–Wallis test, *n* = 5 per each group, Fig. 3A), suggesting a potential direct effect of cladribine on these cells. In contrast, DCK expression on macrophages was significantly decreased at the mRNA level (1.055 ± 0.13 2^−∆∆Ct^ value; *n* = 9; *p* = 0.0421 for macrophages vs. CD19^+^ T cells, *p* = 0.0010 for macrophages vs. neurons, Kruskal–Wallis test), not explaining the significant reduction of macrophages in peripheral blood upon cladribine treatment.

To further assess how cladribine treatment affects dynamic properties of neuronal network activity, we investigated their functionality in acute brain slices. The functional impact of cladribine treatment on the firing patterns of neurons of the primary auditory cortex was investigated by performing single-cell electrophysiology measurements. Cells were recorded under whole-cell current-clamp conditions starting from a membrane potential of − 60 mV and subsequently stimulated using 20 to 160 pA depolarizing current steps (each of 2.5 s duration, with an interval between the steps of 2.0 s). Depolarizing current pulses of increasing amplitude induced incremental numbers of APs in all experimental conditions (Fig. 3C). Neurons from EAE mice treated with vehicle generated more APs in response to the same depolarizing current pulse, compared to control animals (Fig. 3B (left and middle panel) and Fig. 3C). In detail, the vehicle-treated EAE mice demonstrated an increase of approximately 2.5-fold in the number of the generated APs at steps ≥ 100 pA (exemplarily shown at a depolarizing current pulse of 160 pA: control (*n* = 23) vs. EAE-vehicle-treated (*n* = 14): 7.0 ± 1.0 vs. 14.21 ± 2.33 APs; *p* < 0.0001; two-way ANOVA, complemented by Bonferroni test for multiple comparison). The highest number of APs was elicited when current steps ≥ 140 pA were applied.

In contrast, cladribine treatment in EAE significantly reduced the number of APs compared to vehicle-treated EAE mice (exemplarily shown at a depolarization current pulse of 160 pA: cladribine-treated (*n* = 25) vs. vehicle-treated EAE (*n* = 14): 7.24 ± 1.00 vs. 14.21 ± 2.33 APs; *p* < 0.001; two-way ANOVA, complemented by Bonferroni test for multiple comparison, Fig. 3B (middle and right panel) and Fig. 3C). Of note, these effects were not accompanied by differences in resting membrane potential of cortical neurons, nor by their baseline input resistance (control: *n* = 23, vehicle-treated EAE: *n* = 14, cladribine-treated EAE treated: *n* = 24; Fig. 3D). Taken together, these results indicate that EAE induces a hyperactivity of the cortical neuronal network that is normalized by oral cladribine treatment in mice.

To study the characteristics of synaptic transmission, spontaneous EPSCs were recorded in neurons of the primary auditory cortex in voltage-clamp mode. As previously demonstrated [17, 31], the total number of glutamate-mediated EPSCs in neurons recorded over a period of 5 min, increased in the acute phase of EAE (control (*n* = 28) vs. vehicle-treated EAE (*n* = 16): 136.8 ± 24.67 vs. 292.6 ± 57.96 EPSCs; *p* < 0.05; unpaired Mann–Whitney *U* test, Fig. 3E). Interestingly, treatment with oral cladribine completely prevented the alteration of the total number of EPSC (cladribine-treated EAE (*n* = 19): 177.2 ± 30.80; *p* > 0.05 with respect to control mice), indicating an action of this drug at glutamatergic synapses. In contrast, our experiments did not show a change of GABAergic events at *d*_max_ of EAE (control (*n* = 19) vs. vehicle-treated EAE (*n* = 11): 5.0 ± 0.96 vs. 4.46 ± 0.55 IPSCs, *p* > 0.05, unpaired Mann–Whitney *U* test, Fig. 3F), nor an influence of cladribine treatment on GABAergic currents in the cortex (cladribine-treated EAE (*n* = 16): 5.19 ± 1.29 IPSCs; *p* > 0.05 with respect to control mice, unpaired Mann–Whitney* U* test).

Summarized, these data strongly indicate that cladribine has direct neuroprotective effects, by reducing the excitotoxic damage associated with glutamate release in EAE.

### The effects of cladribine are transient at the cellular level

To assess how long lasting the effects of cladribine treatment are on immune cell profile and neuronal functionality networks, we repeated the aforementioned experiments with outcome-measurement at day 27 post-immunization (Fig. 4, Additional file 1: Fig. S1). At this chronic state of EAE, the effects observed by oral cladribine treatment carried out from day 5 to 9 post-immunization are still visible on the clinical EAE score which appeared to be still ameliorated (cladribine-treated (*n* = 12) vs. vehicle-treated (*n* = 13) EAE at d27: 1.96 ± 0.27 vs. 2.77 ± 0.30; *p* < 0.05; unpaired Mann–Whitney* U* test, Fig. 4A). However, distinct immune cell alterations were no longer detectable in any of the organs analyzed in contrast to *d*_max_ (*n* = 3 for each group, cell type and organ; *p* > 0.05 for each cell type, two-way ANOVA, complemented by Bonferroni test for multiple comparisons, Fig. 4B), suggesting a fast immune cell repopulation in our EAE model. In particular, the marked leukocyte depletion in the peripheral blood seen at *d*_max_, recovered to vehicle-control values at day 27 (Fig. 4B). Moreover, immune cell profiles in thymus and LN revealed no differences between the two groups, contrasting the elevated leucocyte (subsets) numbers in these organs following cladribine treatment at *d*_max_ (Fig. 4B). We detected solely an increase of leukocytes in spleens of the cladribine-treated group compared to vehicle-treated animals (cladribine-treated vs. vehicle-treated: 7.01 ± 0.70 vs. 5.12 ± 0.23 *10^6^/mg bw, *p* < 0.0001, Fig. 4B). In the bone marrow, the level of macrophages was also significantly elevated at day 27 (cladribine-treated vs. vehicle-treated: 39.14 ± 4.71 vs. 27.42 ± 1.75% of total cell count, *p* < 0.01, Fig. 4B).

Moreover, immune cell composition in the CNS was similar comparing the two groups, (*p* > 0.05; two-way ANOVA, complemented by Bonferroni test for multiple comparisons, Fig. 4C).

The only effect of cladribine therapy on the CNS immune profile at day 27 post-EAE induction was a reduction in the microglia count in the brains of cladribine-treated EAE mice compared to vehicle-treated animals (cladribine-treated vs. vehicle-treated: 9.32 ± 2.46 vs. 17.51 ± 1.12 *10^4^/ g brain, *p* < 0.0001; Fig. 4C).

To investigate possible reasons for the shorter lasting transient reduction of blood leukocytes by cladribine in mice compared with what is known for MS patients, we compared DCK mRNA expression levels in both species. We appreciated that DCK expression was significantly higher in mice than in human PBMCs (mice vs. human PBMCs: 6.137 ± 0.95 vs. 1.013 ± 0.07 2^−∆∆Ct^ values, *p* < 0.01; Fig. 4D).

Immunological findings were in line with the electrophysiological results obtained at day 27 (Fig. 4E). The firing patterns of neurons of the auditory cortex recorded under current-clamp conditions was no longer associated with hyperexcitability (control (*n* = 23) vs. EAE-vehicle (*n* = 10) at a depolarizing current step of 120 pA: 6.70 ± 1.02 vs. 8.50 ± 3.38, *p* > 0.05; two-way ANOVA, complemented by Bonferroni test for multiple comparison, Fig. 4E).

Cladribine inhibits leukocyte proliferation and shows a tendency to hinder their egress from lymphoid organs in comparison to vehicle-treated controls in vitro.

To address whether cladribine hinders the egress of leukocytes from lymphoid organs, we performed organ cultures of cervical LNs (Additional file 5: Fig. S5) and spleen (Additional file 6: Fig. S6) from naïve C57BL/6 J wild-type mice with and without cladribine treatment in vitro and checked for migration ratios. The selected cladribine concentration of 0.1 µM is consistent with previous studies and represents the concentration found in vivo [32]. Here, Fissolo et al*.* determined the concentration of cladribine in the plasma of patients after administration of a 10 mg tablet and tested its efficacy in in vitro experiments. Further, we characterized the migrated cells by flow cytometry of migration and activation markers (CD25, CD69, CD11a, CD44, CD62L, and CD49d). In both, cervical LN and spleen, we observed a trend towards a decrease in migration of immune cells upon cladribine treatment, but this was not significant (Additional file 5: Fig. S5A, Additional file 6: Fig. S6A). However, there was a significant difference in expression of CD25 by CD4^+^ cells between control and cladribine treatment (Additional file 5: Fig. S5C, Additional file 6: Fig. S6C). This difference was significant in cervical LNs (*p* = 0.011) and even highly significant (*p* = 0.0005) in the spleen. We hypothesize that this shift was caused by an upregulation of migration of CD4^+^CD25^+^ cells, i.e., regulatory T cells (Tregs) or activated CD4 T cells, upon cladribine treatment. Next, to check whether cladribine increases leukocyte proliferation, we performed a proliferation assay using splenocytes from naïve mice, with and without cladribine treatment (Additional file 7: Fig. S7). We found a highly significant inhibition of proliferation after cladribine treatment (vehicle vs. 0.1 M$\upmu$ cladribine: *p* = 0.0002; vehicle vs. 1 M$\upmu$ cladribine: *p* < 0.0001) (Additional file 7: Fig. S7A). An implied dose dependency (0.1 M$\upmu$ vs. 1 M$\upmu$ cladribine) was not significant. Again, CD25^+^ T cells were most affected by cladribine administration (vehicle vs. 0.1 M$\upmu$ cladribine: *p* = 0.0114; vehicle vs. 1 M$\upmu$ cladribine: *p* < 0.0001), providing further evidence that cladribine inhibits proliferation of splenocytes (Additional file 7: Fig. S7B).

Overall, these data indicate that cladribine inhibits proliferation of leukocytes and shows a tendency to hinder their egress from lymphoid organs.

## Discussion

The present study demonstrates that clinical deterioration and synaptic defects of EAE mice can be significantly attenuated by cladribine, suggesting that treatment with this pharmacological agent could exert both, an immunosuppressive as well as a potential neuroprotective effect also in patients with MS.

In our in vivo investigation of the effects of oral cladribine administration, we observed significantly attenuated clinical EAE scores in cladribine- compared to vehicle-treated EAE mice. Both the initial course of deterioration was delayed and the maximum disease symptoms were lowered, indicating functional protection of cladribine in our mouse model of MS. This is consistent with a previous study by Musella et al*.*, which showed that intrathecal cladribine significantly reduces clinical EAE scores [13].

Beneficial clinical effects of cladribine were accompanied by significantly reduced leukocyte subsets in peripheral blood and decreased numbers of infiltrating immune cells in the spinal cord and brain of mice compared to vehicle-treated controls. The spinal cord was more affected, which was expected, as it represents the major lesion site in our EAE model at disease peak [15, 19]. Unexpectedly, we detected an increase of leukocytes in the LN and thymus of cladribine-treated animals as compared to vehicle-treated controls. This might suggest that one mechanism of cladribine-induced improvement of MS symptoms could be a reduced egress of these cells from lymphoid organs. As an alternative, increased proliferation of leucocytes might also occur. Anyway, cladribine effects on lymphatic organs have not yet been described in the literature and require verification in future studies.

Moreover, we observed a significant reduction in innate immune cells counts in the peripheral blood and spinal cord following cladribine treatment. Our data further indicated that cladribine inhibits proliferation of leukocytes and shows a trend to hinder their egress from lymphoid organs, although not significant. In contrast, immunophenotyping studies in humans show that peripheral innate immune system cells are largely unaffected by treatment with cladribine [28–30]. This was consistent with the low levels of enzymatic activity of DCK of these immune cells, determining sensitivity (or resistance) to cladribine toxicity [6]. Whether this difference in susceptibility to cladribine is caused by different expression levels of DCK between humans and mice still needs to be investigated in the future.

Given that cladribine is a small molecule that freely diffuses across the blood–brain barrier, a direct neuroprotective effect can be suggested [8]. Consistent with this hypothesis, our results potentially indicate a direct action of cladribine on pyramidal cortical neurons, as both cellular hyperexcitability of these neurons and changes in glutamatergic synaptic transmission seen in EAE mice were rescued by oral administration of this drug. However, our experiments do not allow us to clearly differentiate whether these findings are attributable to a direct neuroprotective effect of cladribine or simply to the reduction of CNS inflammation.

Of note, based on a series of experimental findings, abnormal glutamate-dependent synaptic excitation has been proposed as a crucial mechanism of MS-induced neurodegeneration [31]. Despite a few controversial results [33], high levels of glutamate have been detected in brains of EAE animals [34, 35], in the cerebrospinal fluid [36, 37] as well as in WM and GM lesions of active MS patients [38]. Synaptic alterations were partially attributed to immune cell–neuron interactions [17, 39]. In this context, the permanent increase in proinflammatory cytokine levels in MS together with the increased availability of glutamate seemed responsible for upregulated neuronal glutamate receptor expression, further reinforcing the local glutamate excitotoxicity [39]. Indeed, pharmacological treatment with glutamate receptor antagonists exhibited beneficial effects in EAE [16, 40, 41] and in a small study of MS patients [42].

In addition, accumulating evidence points to dysregulation of the GABAergic system in both MS [43, 44] and EAE [45–47], though a few contrasting results have been published [48, 49]. Autoimmune CNS inflammation seems to reduce GABA signaling [45, 46], thereby further favoring excitotoxic neuronal damage. Of note, in our experiments reduced GABAergic events did not seem to contribute to the increased excitability of neurons in layer 4 of the auditory cortex since spontaneous GABAergic transmission was unchanged following cladribine treatment.

Taken together, synaptopathy seems an early pathological hallmark of both MS and EAE, with MS-related CNS inflammation likely resulting in unbalanced synaptic hyperactivation, synaptic loss and finally neurodegeneration. Importantly, our results with oral cladribine treatment show that these inflammation-driven synaptic abnormalities are at least partially reversible and, therefore, represent attractive therapeutic targets for MS. Based on this assumption further studies are needed to clarify the mechanisms underlying cladribine effects on synaptic and neuronal function during EAE. Since sustained changes in synaptic plasticity, like long-term potentiation has not been assessed in the present study, potential durable effects of cladribine on neuronal function still need to be identified [50].

Of note, in our experimental setting, effects of cladribine on immune cell alterations and electrophysiological neuronal function were transient, only detectable in the acute stage of the disease (*d*_max_) but not at a chronic state (day 27). This is an unexpected finding, as cladribine has been postulated as a so-called immune reconstitution therapy with the potential to induce long-term or even durable drug-free remission in patients with MS [51]. Accordingly, studies of lymphocyte reduction and repopulation dynamics after cladribine treatment in humans showed that lymphocyte recovery began soon after treatment in years 1 and 2, but it required approximately 30 weeks (for B lymphocytes) and 43 weeks (for T cells) after the last dose of cladribine to reach threshold levels again [30]. One possible explanation of this discrepancy might be that in our experiments we applied a different administration schedule than used in the clinic. While in RMS patients, cladribine is normally administered in two courses of 5 days being 4 weeks apart in year one and two, we only performed one 5-day treatment course. Moreover, cladribine was applied in the pre-acute phase of EAE but was withdrawn before the peak of the disease activity was reached. Therefore, it is plausible to assume that the (harmful) effects of MOG application on the immune system reappeared due to the ongoing active disease process after cessation of treatment. It is therefore more likely that the clinical effect we still see at day 27 post-EAE merely reflects the difference previously induced by cladribine administration. Interestingly, we found that DCK expression was significantly higher in mouse PBMCs compared to human PBMCs. Hence, the hypothesis that higher doses are required and/or activation of the active metabolite is less efficient, does not seem to account for the shorter duration of effects of cladribine on lymphocytes in mice as compared to humans.

## Conclusion

In conclusion, we demonstrated here that oral cladribine attenuates the clinical EAE score in mice. The in vivo effect was accompanied by a compartment-specific immune profile, particularly targeting circulating and brain-infiltrating leukocytes, with little effect on lymphoid organs. Moreover, we observed that cladribine reverted EAE-induced hyperexcitability of neurons from the primary auditory cortex to physiological-like and mitigated the effects of inflammation on spontaneous synaptic transmission in rodents. These results suggest a possible neuroprotective mechanism of cladribine, combined to the known immunosuppressive effect in experimental MS.

## Supplementary Information

**Additional file 1: Figure S1**. Experimental scheme. Active MOG EAE was induced as previously described by immunization (day 0) of C57BL/6J mice with MOG_35–55_ peptide, followed by pertussis toxin (PTX) injections (day 0 and day 2) (15). Mice were divided into two experimental groups: Group 1 received cladribine via oral gavage (10 mg/kg from day 5 to day 9), while group 2 received only the vehicle for the same period of time. On day 10 post-EAE induction, focal EAE lesions were generated by stereotactic injection of proinflammatory cytokines (interferon gamma (INF-γ) and tumor necrosis factor alpha (TNF-α) into the auditory cortex to induce cortical grey matter lesions. Experimental read-out (electrophysiological recordings, flow cytometric immunophenotyping, histology) was performed either on d_max_ (defining the day of maximal clinical deterioration) or on day 27 post-EAE induction (to assess the chronical EAE state).

**Additional file 2: Figure S2**. Titration curves of oral cladribine treatment in mice. In preliminary experiments, we analyzed the plasma [ng/ml] and brain concentrations [ng/g] of oral cladribine treatment with different doses (3.25, 5 and 10 mg/kg bodyweight) over a time period of 2.5 h. Every 30 min blood was drawn and a proportion of animals was killed to obtain brain tissue for titration analyses. Curves show the maximal concentration of cladribine in blood and brain tissue (C_max_ [ng/ml or ng/g]), the time to maximal concentration (t_max_ [hours]) and the area under the curve (AUC [ng*h/ml or ng*h/g]).

**Additional file 3: Figure S3**. Gating strategy for flow cytometry. Single immune cells from the periphery (A, here exemplary shown with spleen tissue) and the central nervous system (CNS; B, here exemplary shown with spinal cord tissue) were simultaneously analyzed by flow cytometry. Total leukocytes were identified by forward scatter (FSC) and sideward scatter (SSC) and cell-doublets were removed by FSC width and FSC height gating. From these cells, we identified leukocytes subsets based on their surface marker expression: CD45R^+^ B cells; CD3^+^CD4^+^ T-helper cells, CD3^+^CD8^+^ cytotoxic T cells and CD4^+^CD8^+^ double positive T cells; CD3^−^NK1.1^+^ natural killer (NK) cells; CD11b^+^CD11c^−^ monocytes and macrophages (M/M) and CD11b^+^CD11c^+^ dendritic cells. Regarding the CNS tissue, prior to discrimination of leukocyte subsets, we differentiated microglia cells (CD45^med^) from infiltrated leukocytes (CD45^high^).

**Additional file 4: Figure S4**. Gating strategy for sorting of macrophages. CD11b^+^ cells were isolated from murine CNS and spinal cord via magnetic beads. Subsequently, CD11b^+^ cells were simultaneously analyzed and sorted by flow cytometry. Total CD11b^+^ cells were identified by forward scatter (FSC) and sideward scatter (SSC) (A), and cell-doublets were removed by SSC width and SSC height gating (B). From these cells, we discriminated macrophages based on their surface marker expression of CD45^high^CD11b^high^ (C). Macrophages were sorted for downstream experiments (D). Plots (E)-(F) show the distribution of microglia (CD45^interm^CD11b^high^) in comparison to macrophages.

**Additional file 5: Figure S5**. Migration assay of leukocytes in organ cultures of cervical LNs from naïve wild-type mice, with and without cladribine treatment in vitro. A: Migration ratios (number of migrated cells: total cell count) of viable (live), CD4^+^ or CD8^+^ T cells and B cells after cladribine treatment (vehicle (migration) versus cladribine 0.1 µM (migration)) (*n* = 6 for both vehicle and cladribine treatment, *two-way ANOVAs*, *p*-values > 0.05). B: Flow cytometric analysis of the immune cell distribution in % in LNs (vehicle or cladribine (organ)) and the migrated immune cells (vehicle or cladribine (migration)) (*n* = 6 for both vehicle and cladribine treatment, *two-way ANOVAs*). C: Mean fluorescence intensities (MFIs) of migration and activation markers in viable CD4^+^ cells still located in the respective LN (vehicle or cladribine (organ)) compared to those after egress (vehicle or cladribine ((migration)) (*n* = 6 for both vehicle and cladribine treatment, *two-way ANOVAs).* D: MFIs of migration and activation markers in viable CD8^+^ cells still located in the respective LN (vehicle or cladribine (organ)) compared to those after egress (vehicle or cladribine ((migration)) (*n* = 6 for both vehicle and cladribine treatment, *two-way ANOVAs*, *p*-values > 0.05*).* E: MFIs of migration and activation markers in viable B cells still located in the respective LN (vehicle or cladribine (organ)) compared to those after egress (vehicle or cladribine ((migration)) (*n* = 6 for both vehicle and cladribine treatment, *two-way ANOVAs).* *p* > 0.05 = ns, *p* < 0.05 = *, *p* < 0.01 = **, *p* < 0.0001 = ****.

**Additional file 6: Figure S6**. Migration assay of leukocytes in organ cultures of spleens from naïve wild-type mice, with and without cladribine treatment in vitro. A: Migration ratios (number of migrated cells: total cell count) of viable (live), CD4^+^ or CD8^+^ T cells and B cells upon cladribine treatment (vehicle (migration) versus cladribine 0.1 µM (migration)) (*n* = 6 for both vehicle and cladribine treatment, *two-way ANOVAs*, *p*-values > 0.05). B: Flow cytometric analysis of the immune cell distribution in % in spleens (vehicle or cladribine (organ)) and the migrated immune cells (vehicle or cladribine (migration)) (*n* = 6 for both vehicle and cladribine treatment, *two-way ANOVAs*). C: Mean fluorescence intensities (MFIs) of migration and activation markers in viable CD4^+^ cells still located in the respective spleen (vehicle or cladribine (organ)) compared to those after egress (vehicle or cladribine ((migration)) (*n* = 6 for both vehicle and cladribine treatment, *two-way ANOVAs).* D: MFIs of migration and activation markers in viable CD8^+^ cells still located in the respective spleen (vehicle or cladribine (organ)) compared to those after egress (vehicle or cladribine ((migration)) (*n* = 6 for both vehicle and cladribine treatment, *two-way ANOVAs*, *p*-values > 0.05*).* E: MFIs of migration and activation markers in viable B cells still located in the respective spleen (vehicle or cladribine (organ)) compared to those after egress (vehicle or cladribine ((migration)) (*n* = 6 for both vehicle and cladribine treatment, *two-way ANOVAs).* *p* > 0.05 = ns, *p* < 0.05 = *, *p* < 0.01 = **, *p* < 0.001 = ***, *p* < 0.0001 = ****.

**Additional file 7: Figure S7**. Proliferation assay of splenocytes from naïve wild-type mice, with and without cladribine treatment in vitro. A: Proliferated cells in % after cladribine treatment (with 0.1 or 1 μM cladribine) versus vehicle-treated cells. We compared the unstimulated to the stimulated setting (stimulated with 1 μg/mL anti-CD3 and 2 μg/mL anti-CD28 for 3 days) (*n* = 6 for both vehicle and cladribine treatment, *two-way ANOVAs*). B: Mean fluorescence intensities (MFIs) of migration and activation markers in all viable stimulated cells upon vehicle- compared to cladribine-treatment (*n* = 6 for both vehicle and cladribine treatment, *two-way ANOVAs).* *p* > 0.05 = ns, *p* < 0.05 = *, *p* < 0.001 = ***, *p* < 0.0001 = ****.

## Data Availability

The datasets used and/or analyzed during the current study are available from the corresponding author on reasonable request. Please contact christinabarbara.schroeter@med.uni-duesseldorf.de.

## Abbreviations

AP: Action potential
ACSF: Artificial cerebrospinal fluid
BW: Bodyweight
CNS: Central nervous system
CFA: Complete Freund`s adjuvant
DCK: Deoxycytidine kinase
EPSC: Excitatory postsynaptic current
EAE: Experimental autoimmune encephalomyelitis
FACS: Fluorescence-activated cell sorting
GM: Grey matter
IPSC: Inhibitory postsynaptic current
IFN-γ: Interferon gamma
LN: Lymph node
MFI: Mean fluorescence intensity
MS: Multiple sclerosis
MOG_35–55_: Myelin oligodendrocyte glycoprotein 35–55
NK cell: Natural killer cell
NKT cell: Natural killer T cell
n.d.: Not detected
ns: Not significant
PBMC: Peripheral blood mononuclear cell
PBS: Phosphate-buffered saline
PTX: Pertussis toxin
qPCR: Quantitative polymerase chain reaction
RMS: Relapsing multiple sclerosis
SEM: Standard error of the mean
TNF-α: Tumor necrosis factor alpha
WM: White matter
