# Supplementary figures and images for: Cladribine treatment improves cortical network functionality in a mouse model of autoimmune encephalomyelitis
Source: J Neuroinflammation. 2022 Nov 8;19:270. doi: 10.1186/s12974-022-02588-7 (PMC9641831; doi:10.1186/s12974-022-02588-7)

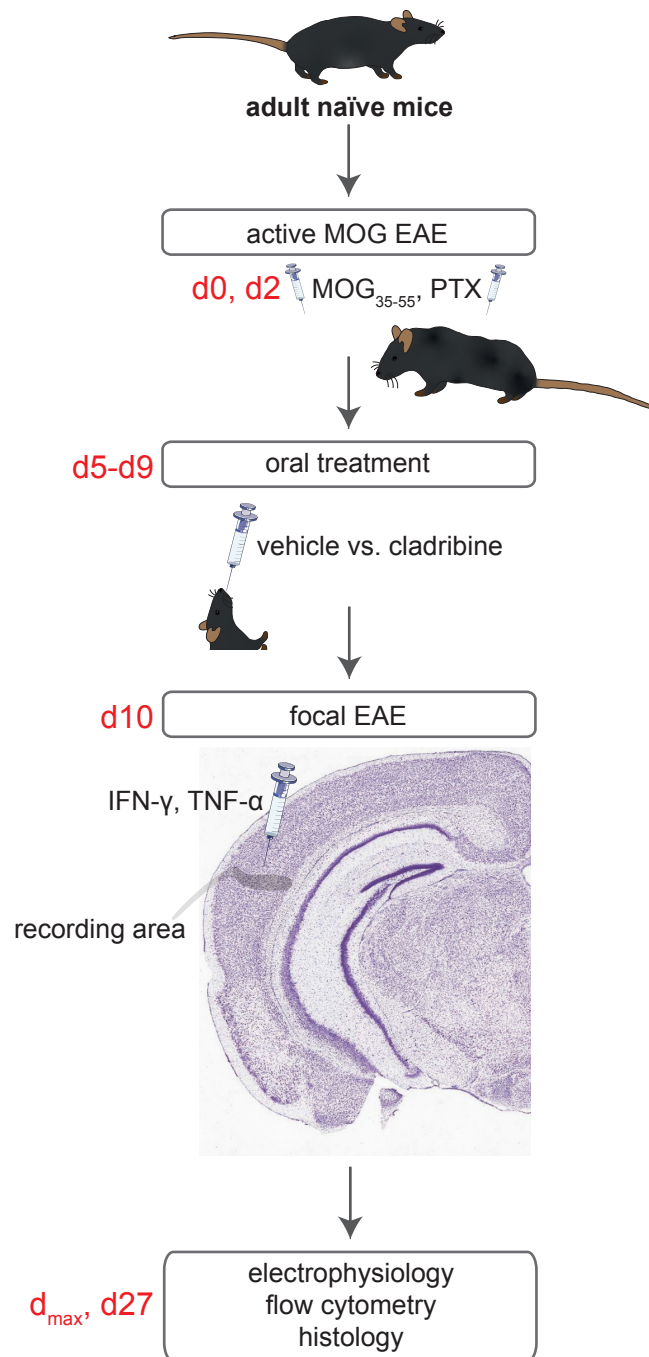

Supplement: Supplementary file 1 — Additional file 1: Figure S1. Experimental scheme. Active MOG EAE was induced as previously described by immunization (day 0) of C57BL/6J mice with MOG35–55 peptide, followed by pertussis toxin (PTX) injections (day 0 and day 2) (15). Mice were divided into two experimental groups: Group 1 received cladribine via oral gavage (10 mg/kg from day 5 to day 9), while group 2 received only the vehicle for the same period of time. On day 10 post-EAE induction, focal EAE lesions were generated by stereotactic injection of proinflammatory cytokines (interferon gamma (INF-γ) and tumor necrosis factor alpha (TNF-α) into the auditory cortex to induce cortical grey matter lesions. Experimental read-out (electrophysiological recordings, flow cytometric immunophenotyping, histology) was performed either on dmax (defining the day of maximal clinical deterioration) or on day 27 post-EAE induction (to assess the chronical EAE state). [file 12974_2022_2588_MOESM1_ESM.pdf]

—●— brain concentration      —●— plasma concentration

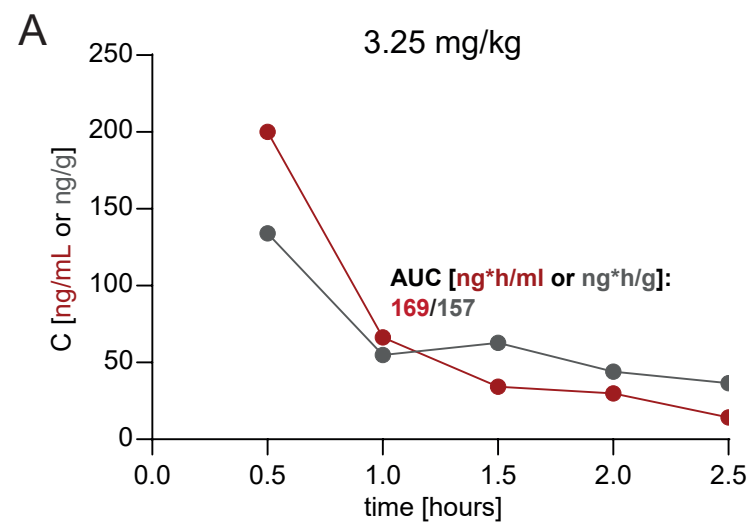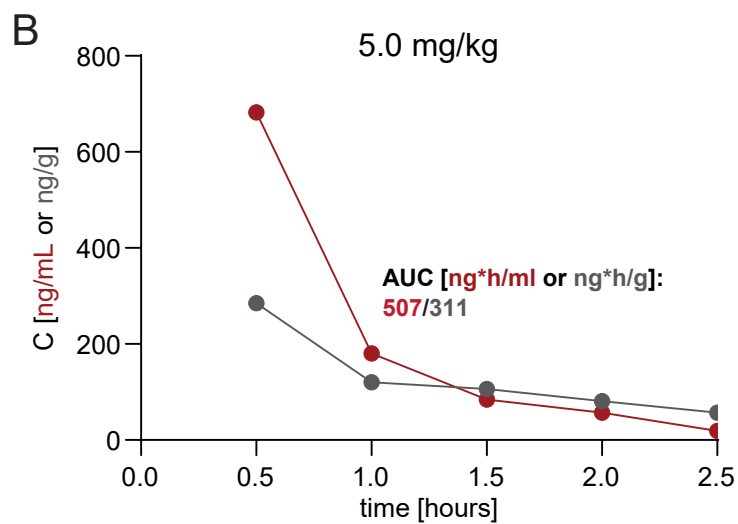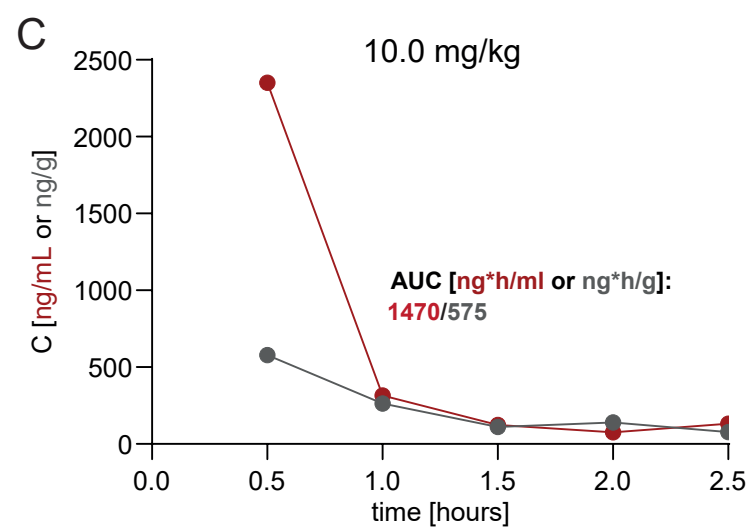

Supplement: Supplementary file 2 — Additional file 2: Figure S2. Titration curves of oral cladribine treatment in mice. In preliminary experiments, we analyzed the plasma [ng/ml] and brain concentrations [ng/g] of oral cladribine treatment with different doses (3.25, 5 and 10 mg/kg bodyweight) over a time period of 2.5 h. Every 30 min blood was drawn and a proportion of animals was killed to obtain brain tissue for titration analyses. Curves show the maximal concentration of cladribine in blood and brain tissue (Cmax [ng/ml or ng/g]), the time to maximal concentration (tmax [hours]) and the area under the curve (AUC [ng*h/ml or ng*h/g]). [file 12974_2022_2588_MOESM2_ESM.pdf]

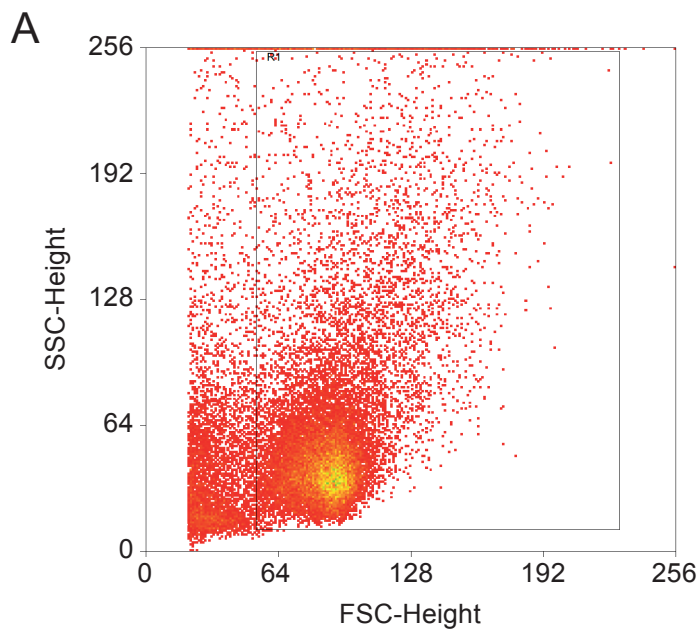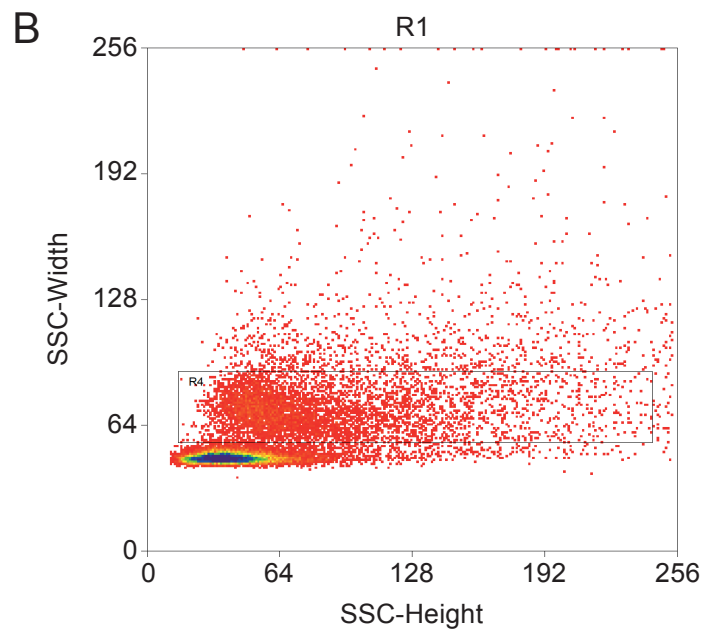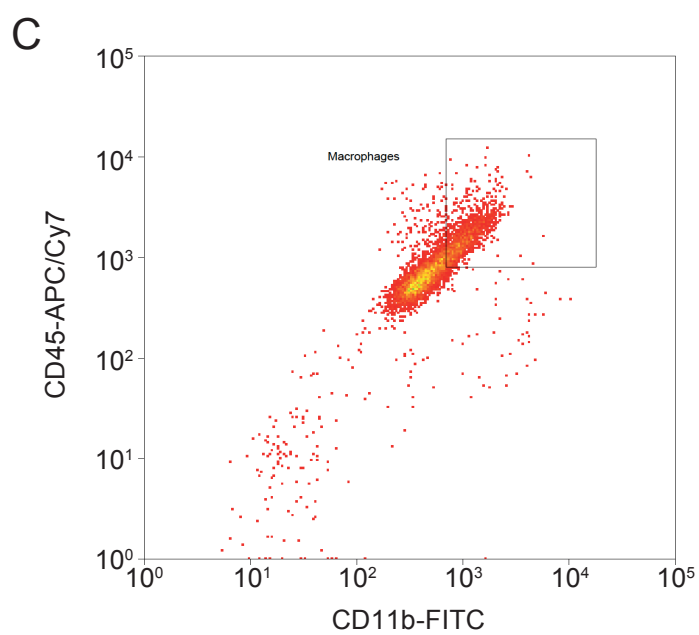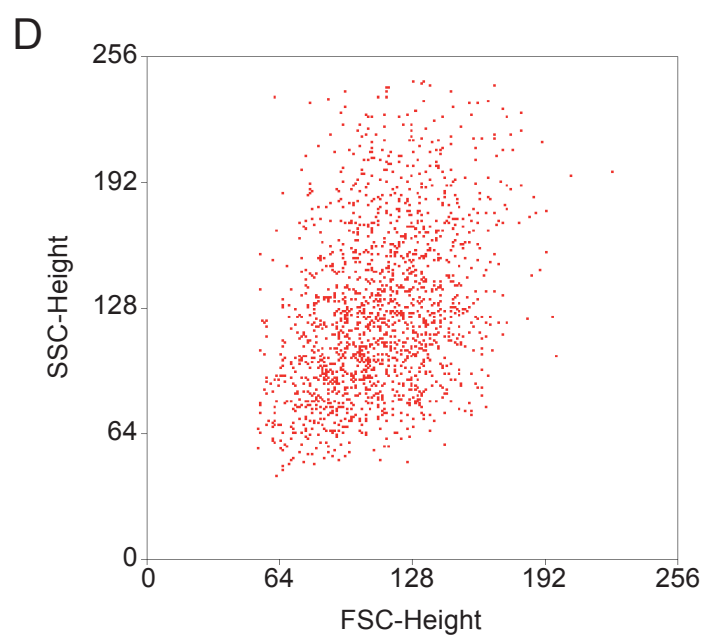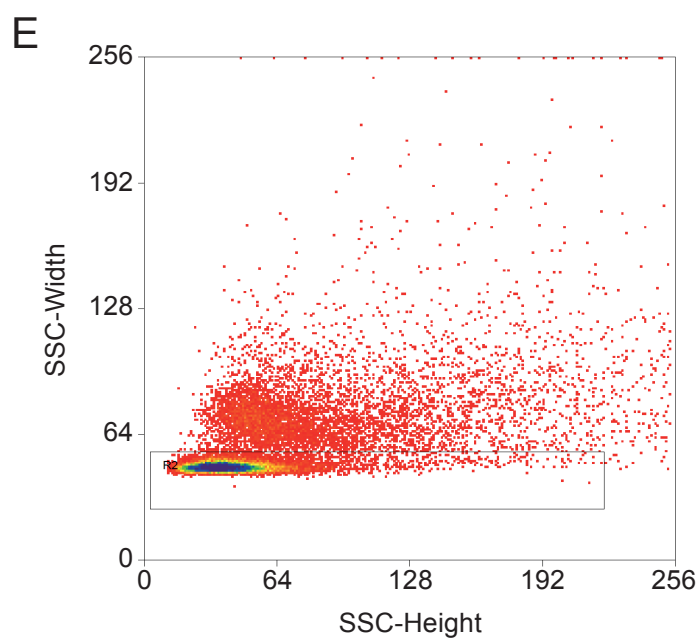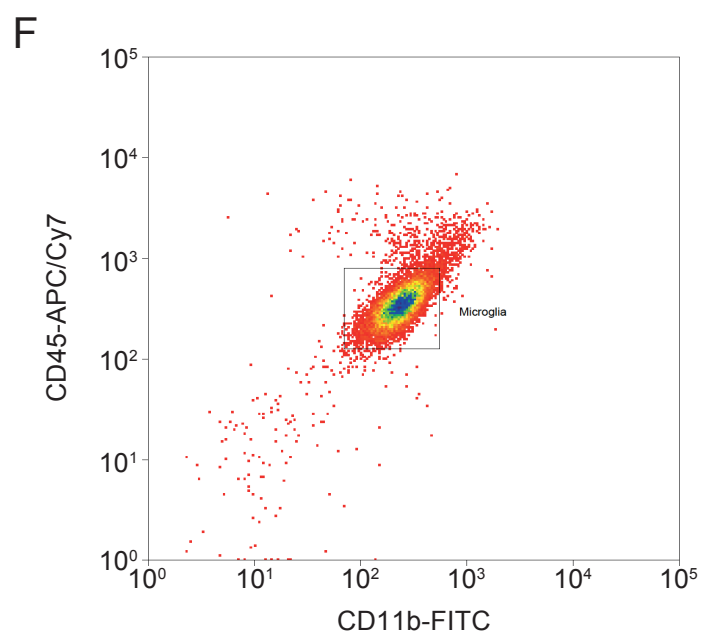

Supplement: Supplementary file 4 — Additional file 4: Figure S4. Gating strategy for sorting of macrophages. CD11b+ cells were isolated from murine CNS and spinal cord via magnetic beads. Subsequently, CD11b+ cells were simultaneously analyzed and sorted by flow cytometry. Total CD11b+ cells were identified by forward scatter (FSC) and sideward scatter (SSC) (A), and cell-doublets were removed by SSC width and SSC height gating (B). From these cells, we discriminated macrophages based on their surface marker expression of CD45highCD11bhigh (C). Macrophages were sorted for downstream experiments (D). Plots (E)-(F) show the distribution of microglia (CD45intermCD11bhigh) in comparison to macrophages. [file 12974_2022_2588_MOESM4_ESM.pdf]

■ vehicle (organ)   
 ▲ vehicle (migration)   
 ■ cladribine (organ)   
 ▲ cladribine (migration)

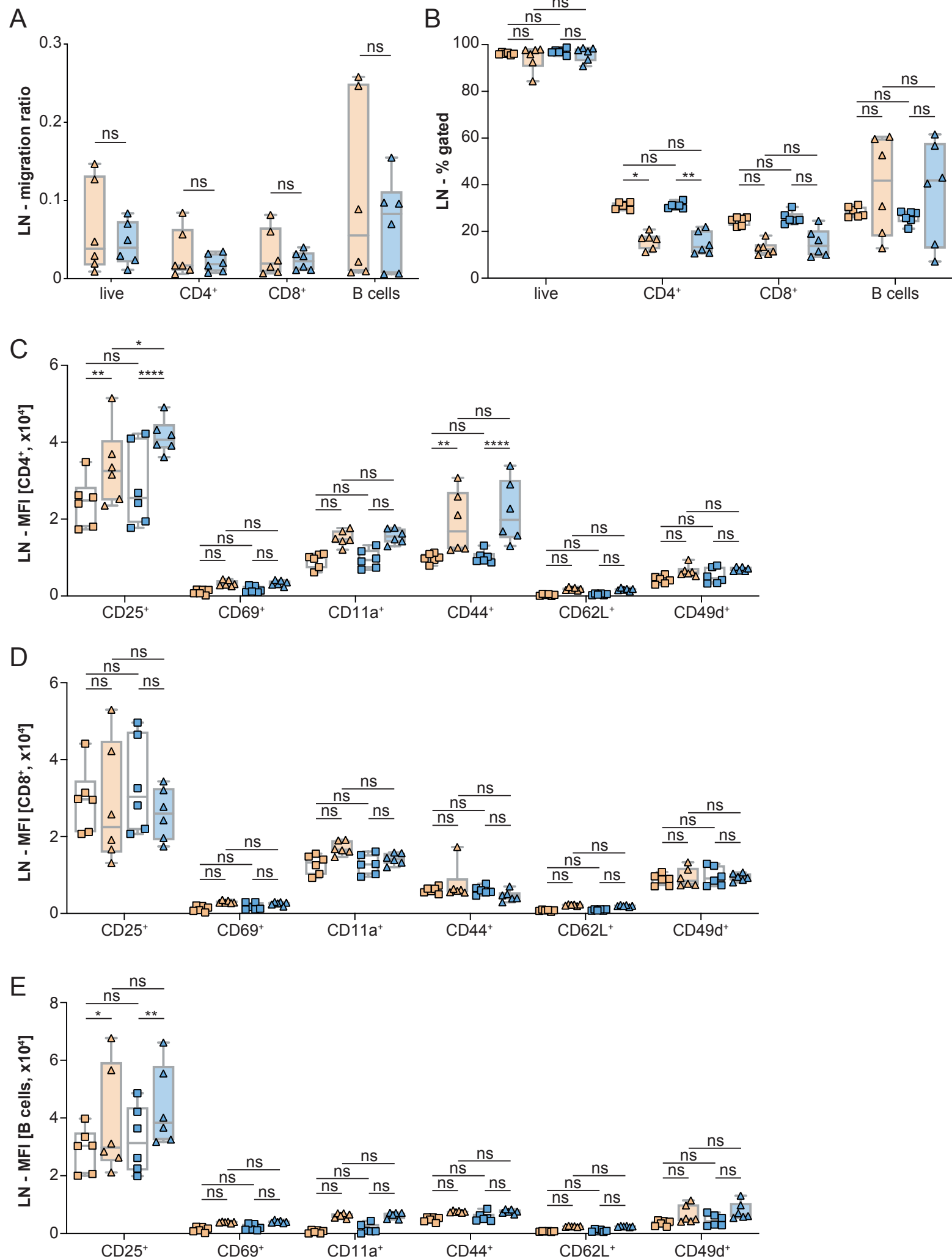

Supplement: Supplementary file 5 — Additional file 5: Figure S5. Migration assay of leukocytes in organ cultures of cervical LNs from naïve wild-type mice, with and without cladribine treatment in vitro. A: Migration ratios (number of migrated cells: total cell count) of viable (live), CD4+ or CD8+ T cells and B cells after cladribine treatment (vehicle (migration) versus cladribine 0.1 µM (migration)) (n = 6 for both vehicle and cladribine treatment, two-way ANOVAs, p-values > 0.05). B: Flow cytometric analysis of the immune cell distribution in % in LNs (vehicle or cladribine (organ)) and the migrated immune cells (vehicle or cladribine (migration)) (n = 6 for both vehicle and cladribine treatment, two-way ANOVAs). C: Mean fluorescence intensities (MFIs) of migration and activation markers in viable CD4+ cells still located in the respective LN (vehicle or cladribine (organ)) compared to those after egress (vehicle or cladribine ((migration)) (n = 6 for both vehicle and cladribine treatment, two-way ANOVAs). D: MFIs of migration and activation markers in viable CD8+ cells still located in the respective LN (vehicle or cladribine (organ)) compared to those after egress (vehicle or cladribine ((migration)) (n = 6 for both vehicle and cladribine treatment, two-way ANOVAs, p-values > 0.05). E: MFIs of migration and activation markers in viable B cells still located in the respective LN (vehicle or cladribine (organ)) compared to those after egress (vehicle or cladribine ((migration)) (n = 6 for both vehicle and cladribine treatment, two-way ANOVAs). p > 0.05 = ns, p < 0.05 = *, p < 0.01 = **, p < 0.0001 = ****. [file 12974_2022_2588_MOESM5_ESM.pdf]

■ vehicle (organ)   
 ▲ vehicle (migration)   
 ■ cladribine (organ)   
 ▲ cladribine (migration)

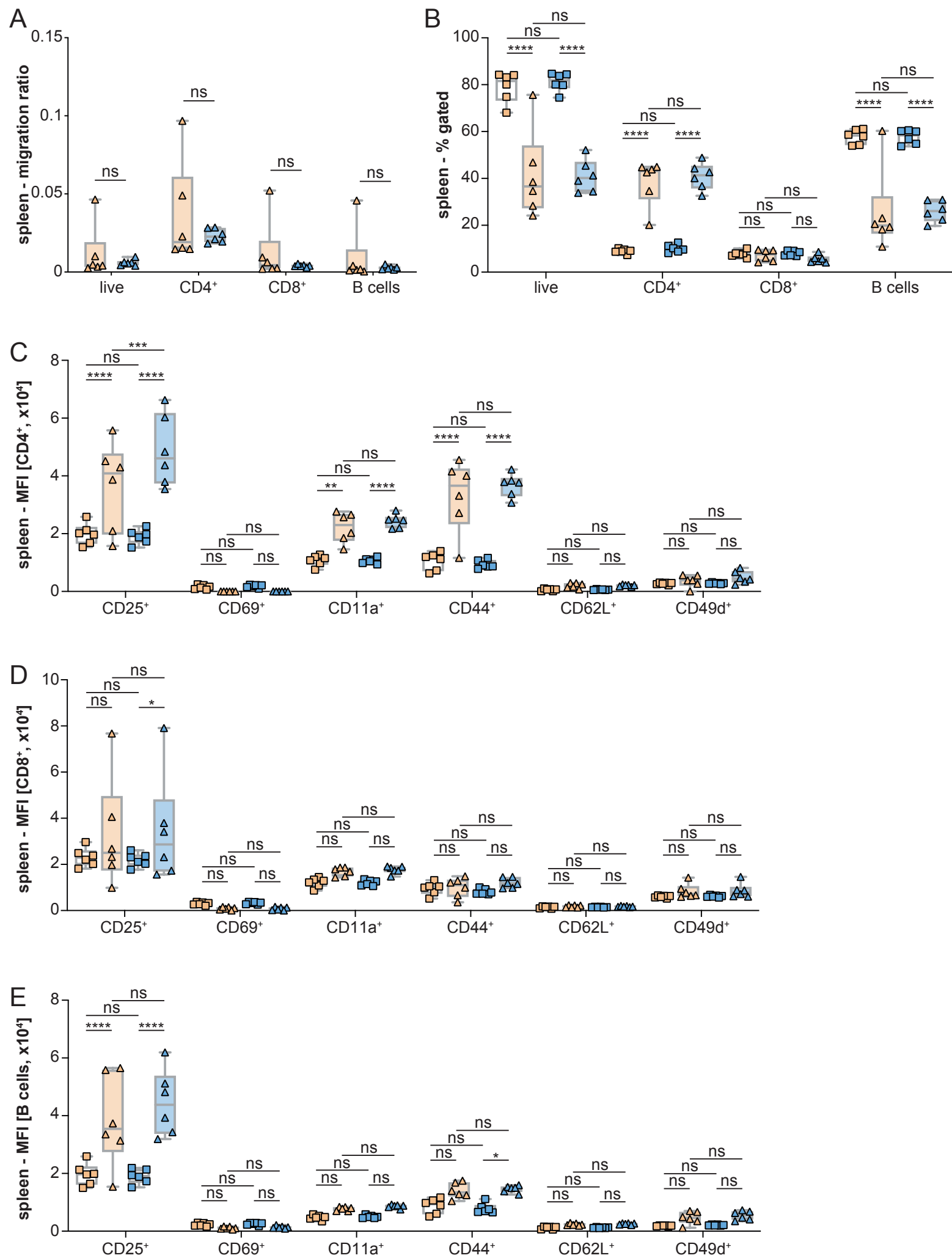

Supplement: Supplementary file 6 — Additional file 6: Figure S6. Migration assay of leukocytes in organ cultures of spleens from naïve wild-type mice, with and without cladribine treatment in vitro. A: Migration ratios (number of migrated cells: total cell count) of viable (live), CD4+ or CD8+ T cells and B cells upon cladribine treatment (vehicle (migration) versus cladribine 0.1 µM (migration)) (n = 6 for both vehicle and cladribine treatment, two-way ANOVAs, p-values > 0.05). B: Flow cytometric analysis of the immune cell distribution in % in spleens (vehicle or cladribine (organ)) and the migrated immune cells (vehicle or cladribine (migration)) (n = 6 for both vehicle and cladribine treatment, two-way ANOVAs). C: Mean fluorescence intensities (MFIs) of migration and activation markers in viable CD4+ cells still located in the respective spleen (vehicle or cladribine (organ)) compared to those after egress (vehicle or cladribine ((migration)) (n = 6 for both vehicle and cladribine treatment, two-way ANOVAs). D: MFIs of migration and activation markers in viable CD8+ cells still located in the respective spleen (vehicle or cladribine (organ)) compared to those after egress (vehicle or cladribine ((migration)) (n = 6 for both vehicle and cladribine treatment, two-way ANOVAs, p-values > 0.05). E: MFIs of migration and activation markers in viable B cells still located in the respective spleen (vehicle or cladribine (organ)) compared to those after egress (vehicle or cladribine ((migration)) (n = 6 for both vehicle and cladribine treatment, two-way ANOVAs). p > 0.05 = ns, p < 0.05 = *, p < 0.01 = **, p < 0.001 = ***, p < 0.0001 = ****. [file 12974_2022_2588_MOESM6_ESM.pdf]

■ vehicle 
 ● cladribine (0.1  $\mu$ M) 
 ▲ cladribine (1  $\mu$ M)

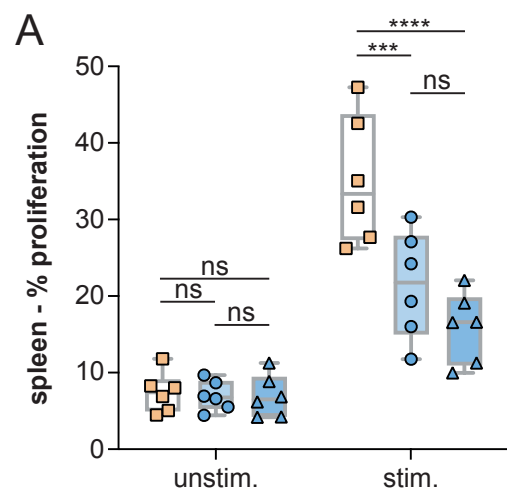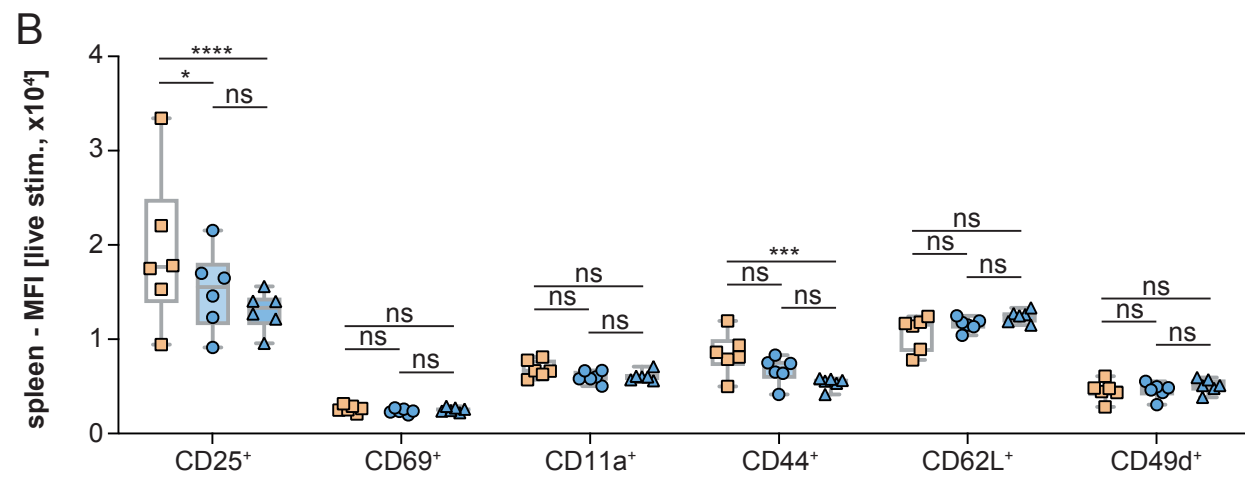

Supplement: Supplementary file 7 — Additional file 7: Figure S7. Proliferation assay of splenocytes from naïve wild-type mice, with and without cladribine treatment in vitro. A: Proliferated cells in % after cladribine treatment (with 0.1 or 1 μM cladribine) versus vehicle-treated cells. We compared the unstimulated to the stimulated setting (stimulated with 1 μg/mL anti-CD3 and 2 μg/mL anti-CD28 for 3 days) (n = 6 for both vehicle and cladribine treatment, two-way ANOVAs). B: Mean fluorescence intensities (MFIs) of migration and activation markers in all viable stimulated cells upon vehicle- compared to cladribine-treatment (n = 6 for both vehicle and cladribine treatment, two-way ANOVAs). p > 0.05 = ns, p < 0.05 = *, p < 0.001 = ***, p < 0.0001 = ****. [file 12974_2022_2588_MOESM7_ESM.pdf]
