# Supplementary material for: Cladribine treatment improves cortical network functionality in a mouse model of autoimmune encephalomyelitis
Source: J Neuroinflammation. 2022 Nov 8;19:270. doi: 10.1186/s12974-022-02588-7 (PMC9641831; doi:10.1186/s12974-022-02588-7)
Supplement: Supplementary file 3 — Additional file 3: Figure S3. Gating strategy for flow cytometry. Single immune cells from the periphery (A, here exemplary shown with spleen tissue) and the central nervous system (CNS; B, here exemplary shown with spinal cord tissue) were simultaneously analyzed by flow cytometry. Total leukocytes were identified by forward scatter (FSC) and sideward scatter (SSC) and cell-doublets were removed by FSC width and FSC height gating. From these cells, we identified leukocytes subsets based on their surface marker expression: CD45R+ B cells; CD3+CD4+ T-helper cells, CD3+CD8+ cytotoxic T cells and CD4+CD8+ double positive T cells; CD3−NK1.1+ natural killer (NK) cells; CD11b+CD11c− monocytes and macrophages (M/M) and CD11b+CD11c+ dendritic cells. Regarding the CNS tissue, prior to discrimination of leukocyte subsets, we differentiated microglia cells (CD45med) from infiltrated leukocytes (CD45high). [file 12974_2022_2588_MOESM3_ESM.pdf]

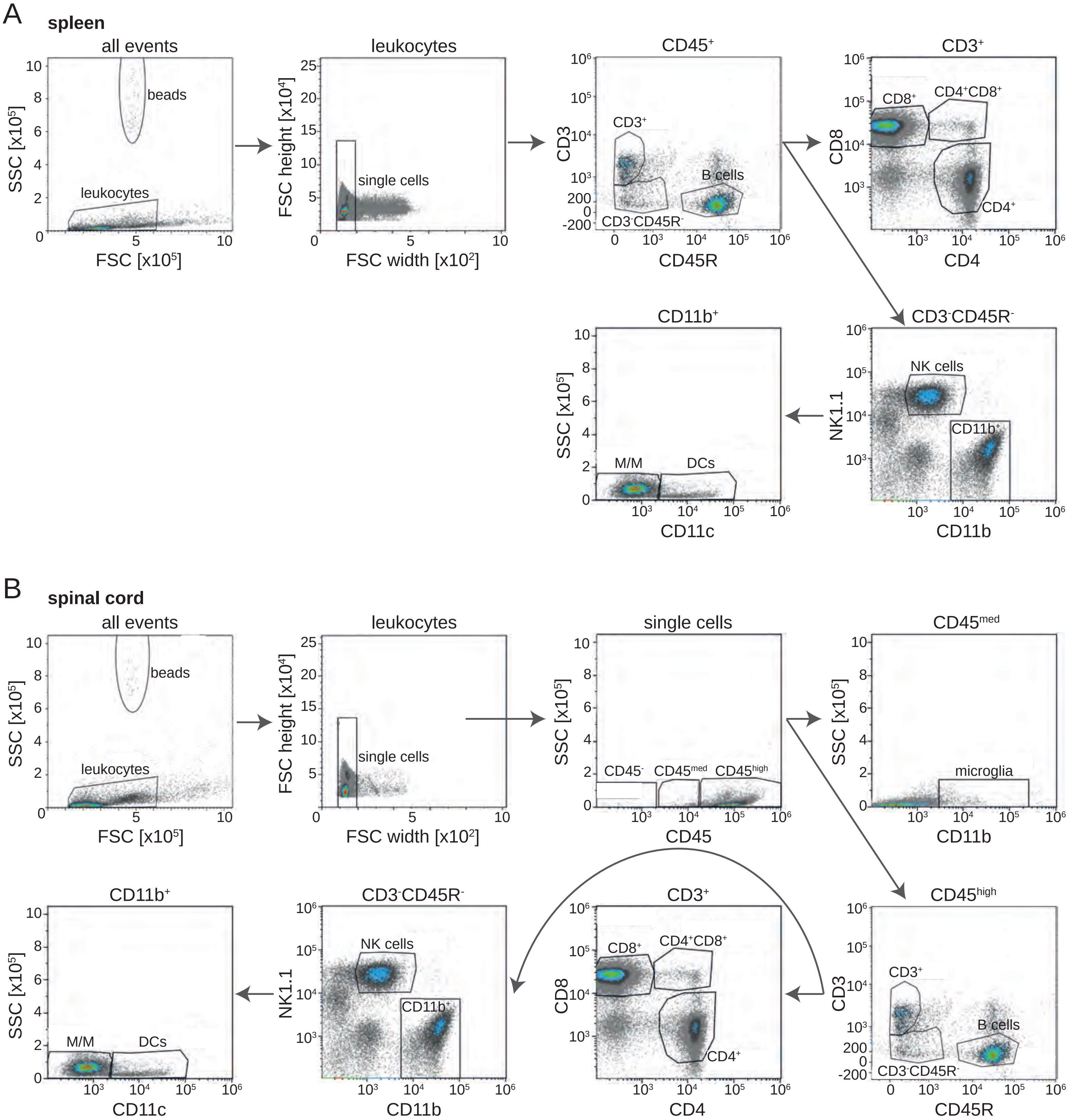
A
spleen
all events
beads
leukocytes
SSC [x10^5]
FSC [x10^5]
leukocytes
single cells
FSC height [x10^4]
FSC width [x10^2]
CD45+
CD3+
B cells
CD3-CD45R-
CD3
CD45R
CD3+
CD8+
CD4+CD8+
CD4+
CD8
CD4
CD3-CD45R-
NK cells
CD11b+
NK1.1
CD11b
CD11b+
M/M
DCs
SSC [x10^5]
CD11c
B
spinal cord
all events
beads
leukocytes
SSC [x10^5]
FSC [x10^5]
leukocytes
single cells
FSC height [x10^4]
FSC width [x10^2]
single cells
CD45-
CD45med
CD45high
SSC [x10^5]
CD45
CD45med
microglia
SSC [x10^5]
CD11b
CD11b+
M/M
DCs
SSC [x10^5]
CD11c
CD3-CD45R-
NK cells
CD11b+
NK1.1
CD11b
CD3+
CD8+
CD4+CD8+
CD4+
CD8
CD4
CD45high
CD3+
B cells
CD3-CD45R-
CD3
CD45R
